# Microscale patchiness controls resource acquisition in populations of foraging bacteria

**DOI:** 10.1126/sciadv.aea5399

**Published:** 2026-11-13

**Authors:** Francesco Carrara, Douglas R. Brumley, Andrew M. Hein, Timothy Cooper, M. Mehdi Salek, Johannes Keegstra, Simon A. Levin, Roman Stocker

**Affiliations:** ^1^ Institute of Environmental Engineering, Department of Civil, Environmental, and Geomatic Engineering, ETH Zurich, 8093 Zurich, Switzerland.; ^2^ School of Mathematics and Statistics, The University of Melbourne, Parkville 3010, Victoria, Australia.; ^3^ Department of Computational Biology, Cornell University, Ithaca, NY 14850, USA.; ^4^ New Engineering Education Transformation, School of Engineering, Massachusetts Institute of Technology, Cambridge, MA 02139, USA.; ^5^ Department of Biological Engineering, Massachusetts Institute of Technology, Cambridge, MA 02139, USA.; ^6^ Department of Ecology and Evolutionary Biology, Princeton University, Princeton, NJ 08544, USA.

## Abstract

Chemotaxis, the ability of cells to detect and respond to chemical gradients, allows them to acquire nutrients, seek hosts, or escape toxins. However, directly measuring bacterial foraging in spatially structured nutrient environments has proven elusive. We developed a new approach to investigate bacterial foraging, on timescales from milliseconds to hours, when exposed to nutrient fluctuations and microscale patchiness mimicking realistic ocean conditions. We generated dynamic, patchy, nutrient landscapes in a large microfluidic arena and simultaneously tracked individual cells of the marine bacterium *Vibrio anguillarum*. Analyzing single-cell nutrient exposure for tens of thousands of cells revealed that the nutrient exposure and distribution of exposure among bacteria depend strongly on the spatiotemporal characteristics of the patchy landscape. Furthermore, bacterial trajectories revealed a progressive decrease of swimming speed counter-balanced by an increase in chemotactic precision, resulting in stable nutrient exposure over time. This work furthers our understanding of bacterial foraging strategies under ecologically relevant conditions.

## INTRODUCTION

One of the fundamental questions in ecology is how environmental fluctuations and patchiness affect the behavior and productivity of populations (*1*–*3*). Theoretical and empirical studies have shown that these fluctuations—spatial, temporal, or both—can have profound effects on the persistence (*4*) and functioning of populations and communities (*5*, *6*). Most studies to date have focused on plants and animals, and much less is known for microorganisms in this respect, including for the model bacterium *Escherichia coli* (*3*). The microenvironments in which bacteria live are often characterized by spatial and temporal variability and resource patchiness. The major impediment for determining how this patchiness influences microbial populations has been the challenge in experimentally recreating structured landscapes and measuring the behavioral response of microorganisms for extended periods of time. Inferring macroscopic nutrient flows in diverse environmental settings, from the gut to soil and the ocean, hinges on the ability to quantify the dependence of microbial foraging on the spatial and temporal heterogeneity in nutrient concentration (*3*, *7*).

Heterotrophic bacteria are the primary consumers of dissolved organic matter (DOM) in the ocean, which they often encounter as a dynamic chemical landscape made of patches, pulses, filaments, and plumes from sinking marine particles (*8*). Many marine bacteria rely on chemotaxis (*9*)—the process of detecting chemical gradients and modifying motility in response—to navigate complex chemical landscapes and to exploit such ephemeral nutrient sources. These microscale processes determine the macroscale kinetics of interactions between microbes and their resources (*10*–*12*) and thereby regulate nutrient flows and set the efficiency of the biological carbon pump (*13*, *14*).

Past experiments have typically studied bacterial chemotaxis in controlled, stable gradients of intermediate to high nutrient concentrations (*15*, *16*), or step-changes in concentration (*17*) and opposing attractant and nutrient gradients (*18*). However, in natural environments such as the coastal ocean, turbulent mixing and molecular diffusion quickly dissipate microscale chemical gradients (*19*), and background concentrations of DOM are often highly dilute, in the range of hundreds of nanomolar to low micromolar (*7*). Recent advances in microfluidics and microfabrication technologies (*20*, *21*), including three-dimensional (3D) printing and the embedding of hydrogels or membranes to create hybrid devices (*22*, *23*), together with the implementation of photolysis of caged compounds (*24*), have dramatically enhanced our capacity to mimic natural settings within controlled microenvironments. This offers an unprecedented ability to study chemotaxis (*25*) and the long-term foraging behavior of microorganisms under conditions representative of the ones they encounter in the wild.

Here, we experimentally show that the spatiotemporal heterogeneity of nutrient availability at the microscale, that is, patchiness, controls spatial clustering in the marine bacterium *Vibrio anguillarum*, and we theoretically predict that this exerts an important control on the efficiency of remineralization. To directly investigate the effect of spatial heterogeneity and temporal fluctuations on long-term chemotactic behavior and population-scale nutrient exposure, we developed a microfluidic method to generate patchy and dynamic chemical landscapes (Fig. 1A). In this approach, we use photolysis of a caged amino acid in a large (1 cm^2^) experimental arena to create a sequence of localized chemoattractant pulses, each becoming available suddenly (as, for example, in a phytoplankton cell lysis event) at the microscale, before diffusing away. Nutrient patchiness is experimentally modulated by changing the frequency of injection of chemical pulses, which results in a different patch density (and, at the same time, a different background concentration). In each patchy landscape, bacterial cells are tracked over several hours to quantify the chemotactic response, the bacterial accumulation, and the nutrient exposure of cells and thus the population. By adapting a mathematical model from the theory of sensory noise (*26*, *27*) to expand the region of parameter space over that explored experimentally, we then quantified the efficiency of bacterial remineralization as a function of microscale nutrient patchiness. Moreover, the capacity to quantify bacterial behavior in realistic, dynamic environments for extended periods of time enabled investigation of the feedback between cell behavior, nutrient exposure, and the chemical microenvironment.

**Fig. 1.**
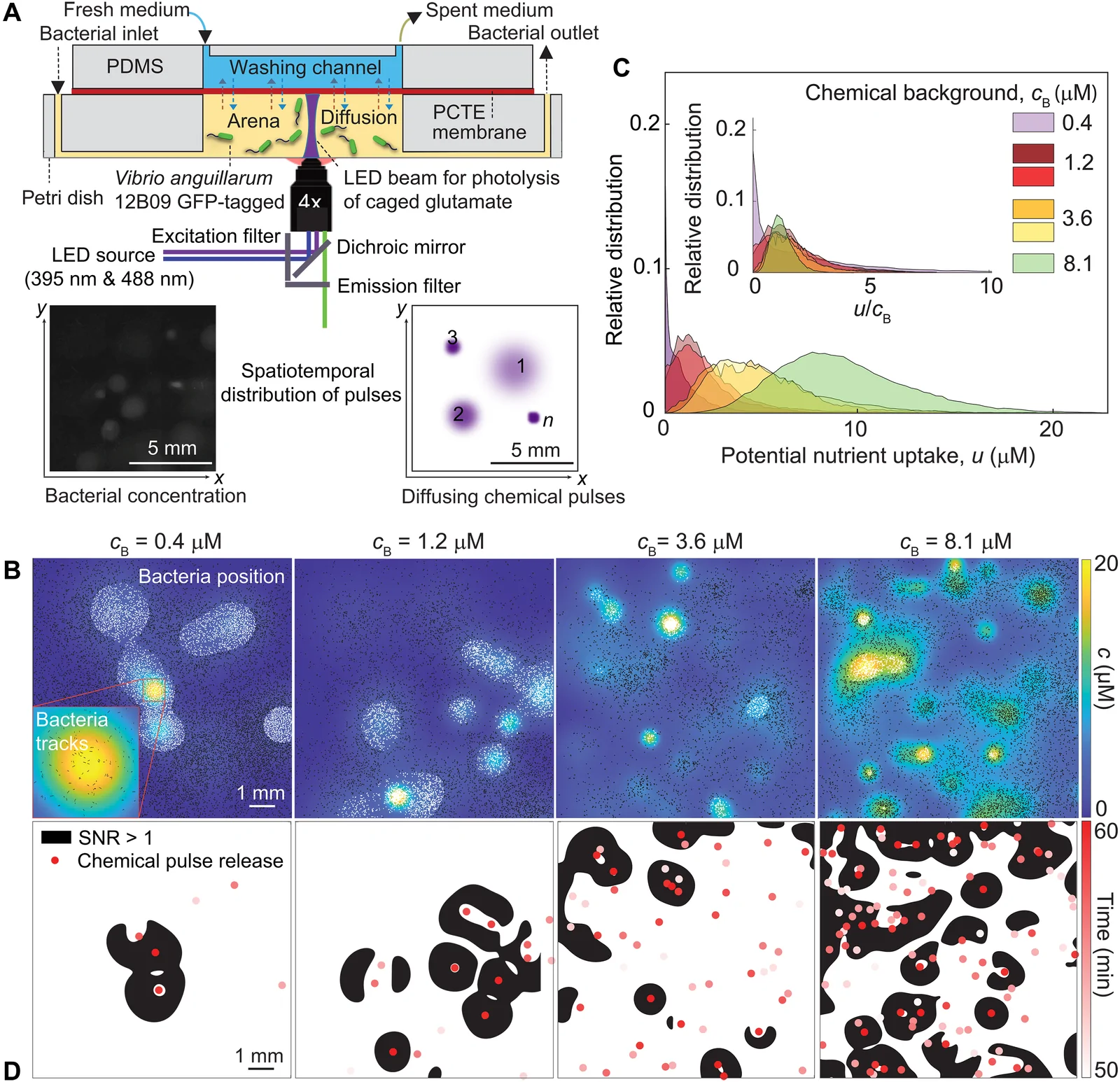
Microscale spatiotemporal heterogeneity of chemoattractant concentration drives bacterial accumulation dynamics in a population of the marine bacterium *Vibrio anguillarum*. (**A**) Schematic of the microfluidic device for creating long-term dynamic nutrient conditions. To create resource landscapes with prescribed spatial and temporal variability, chemical pulses of the chemoattractant glutamate were released at computer-controlled points in time and space by photolysis of caged glutamate within a PDMS microfluidic chamber containing a suspension of GFP-tagged *V. anguillarum*. (**B**) *V. anguillarum* can actively climb transient chemical gradients for different levels of nutrient patchiness. A 20-fold range of the chemical background concentration *c*_B_ was studied, from *c*_B_ = 0.4 to 8.1 μM, by increasing the temporal frequency of pulses. Bacterial movement at the single-cell level (magnified bacterial tracks, inset) and accumulation at the population level (dots represent individual bacteria) are obtained every 15 to 30 min over several hours (here shown after ∼1 hour) and superimposed on the chemoattractant concentration (background color). Bacteria are color-coded based on their potential nutrient uptake, i.e., the instantaneous nutrient concentration experienced relative to the background concentration (black, *u* < 2*c*_B_; white, *u* > 2*c*_B_). (**C**) Distribution of potential uptake for a population of cells computed for nutrient landscapes having different patch densities (through varying *c*_B_). The potential uptake for bacterium *i* at position (*x_i_*,*y_i_*) is computed by determining the chemoattractant concentration at that location *c*(*x_i_*,*y_i_*,*t*) at time *t* from numerical reconstructions. Inset: Distribution of potential uptake rescaled by *c*_B_. (**D**) Binary maps of the instantaneous signal-to-noise ratio (SNR) corresponding to the nutrient profiles in (B), with the position of the chemical pulses released in the previous 10 min superimposed (red-tone dots, with color representing time of pulse release). Black patches indicate SNR(*x*,*y*) > 1, corresponding to the regions where *V. anguillarum* can perform chemotaxis (see the Supplementary Materials).

We found that at high patchiness, the ability of bacteria to climb gradients, and therefore to increase overall nutrient acquisition by the population, increases linearly with increasing nutrient injection rate. This is because when resources are sparse, patches are typically nonoverlapping, and the addition of a new patch results in a proportionally larger region where chemotaxis can occur. Conversely, for landscapes with lower patchiness, the potential uptake begins to saturate. In this situation, where patches are more likely to overlap, addition of more patches causes a comparatively lower increase in the area that elicits a chemotactic response.

## RESULTS

### Bacterial foraging in dynamic, patchy seascapes

Our experiments show that the chemotactic bacterium *V. anguillarum* [previously known as *Vibrio ordalii*; (*28*)] can cluster around diffusing nutrient sources in chemical landscapes characterized by different levels of patchiness and a 20-fold range in background concentrations *c*_B_ (0.4 to 8.1 μM glutamate; Fig. 1B) that are ecologically relevant for coastal seawater (*29*). Here, we define the chemical background *c*_B_ to be the average ambient concentration of the chemoattractant throughout the experimental domain. In each chemical landscape we generated, the swimming speed at the single-cell level and the spatial distribution of cells at the population level were measured every 15 to 30 min over the course of several hours, for tens of thousands of cells. These data allowed us to quantify bacterial accumulation as a function of the dynamic chemoattractant profiles, for different degrees of patchiness.

To study long-term foraging in resource landscapes characterized by microscale patchiness with prescribed spatial and temporal variability, we developed a computer-controlled system to generate, using photolysis, hours-long sequences of pulses released at different random locations within the experimental arena and with controlled intensities, while simultaneously tracking green fluorescent protein (GFP)–tagged bacteria by epifluorescence video microscopy. Molecules of the chemoattractant, glutamate, are caged by photoremovable protecting groups. The caged molecules are added uniformly to the quasi-2D experimental arena (1 cm width by 1 cm length by 500 μm height; area *A* = 1 cm^2^) containing the bacterial suspension, but are not available for bacterial sensing or uptake until their sudden release in a chemical pulse, which occurs at user-defined points in time and space by instantaneously turning on a light-emitting diode (LED) source (Fig. 1A) (*23*). A chemical pulse can be accurately represented by a diffusive cylindrical source [glutamate diffusion coefficient *D* = 608 μm^2^ s^−1^, as previously measured; (*26*)]. The number of molecules released in each pulse is determined by a calibration relationship between exposure time and uncaging fraction (Materials and Methods). In our experiments, this is fixed at a mass (number of molecules per unit height in the chamber) of M* = 2.3 × 10^9^ μm^−1^. Microscale nutrient pulses are created periodically over the 1-cm^2^ arena with a fixed interval Δ*t* between pulses, which we varied across experiments between 5 and 105 s. The resulting total nutrient flux into the arena was thus ϕ = M*/(*A*Δ*tN*_A_) (with units of nanomolar per second), where *N*_A_ is Avogadro’s number. Based on the known pulse locations and sizes, the complete chemical landscape can be readily computed at every time point using the diffusion equation and superimposed with the real-time positions of all tracked bacteria.

The chemoattractant concentration profile *c*(*x*,*y*,*t*) over the experimental arena was determined numerically by solving the diffusion equation with cylindrically symmetric sources in time and space corresponding to those created in the experiments (Materials and Methods). In all numerical simulations used for the reconstruction of the nutrient field, we performed the following approximations: (i) the diffusion process subsequent to the uncaging event of the axisymmetric cylindrical pulse was approximated by a point source spreading with Gaussian profile; (ii) we neglected nutrient consumption by bacteria because cells at low concentrations (*c*_bact_ ≈ 1 × 10^7^ cells ml^−1^) would not substantially affect the local nutrient profile by consumption (see the Supplementary Materials). Numerical simulations in previous work revealed that even diffusion-limited uptake at the same bacterial density does not have an appreciable effect on the chemotactic response (*26*).

To account for the fact that in the ocean, nutrients are also consumed by resident populations, including uniformly distributed nonmotile bacteria, we included in the microfluidic device a washing microchannel directly adjacent to the test arena and separated from it by a nanoporous polycarbonate membrane (PCTE; pore size, 0.4 μm). In this way, a constant flow of fresh medium in the washing channel removes uncaged glutamate and bacterial metabolites from the arena (with a washing timescale τ = 19.5 min), preventing their accumulation, while at the same time replenishing the arena with caged glutamate to permit longer-term experiments (fig. S1). This procedure of nonequilibrium steady state maintains a stable nutrient background, *c*_B_ = ϕτ, over time (after a time *t* ∼ τ; fig. S2A), for different levels of patchiness, thus facilitating long-term experiments.

The patchiness in each experimental landscape can be described by the coefficient of variation CV = <σ_Α_/*c*_B_ >*_t_* of the nutrient in the arena (where the standard deviation σ_Α_ is calculated over the area *A* of the arena) averaged over time (angular brackets), because the process of nutrient release and washing is stationary (fig. S2, B and C).

Our results show that nutrient patchiness drives transient bacterial accumulation and nutrient uptake dynamics in *V. anguillarum* (Fig. 1). We used here the potential uptake (i.e., nutrient exposure) as a proxy of bacterial foraging uptake, a method that has proven accurate in other model systems involving chemotaxis of marine bacteria (*30*). The potential nutrient uptake is computed by determining all the bacterial positions (*x_i_*,*y_i_*,*t*) obtained from a video at time *t*, computing the nutrient concentration *c*(*x_i_*,*y_i_*,*t*) at each position (“nutrient exposure”) and then averaging the nutrient exposure over all cells, *u_t_* = <*c*(*x_i_*,*y_i_*,*t*)>*_i_* (angular brackets denote averaging). Our results show that the nutrient uptake increases overall as a function of patch density, as a direct consequence of the higher nutrient flux and chemical background (Fig. 1C). The proportion of cells in the population that are strongly accumulated around patches is defined as the percentage of cells with potential uptake greater than twice the background (*u_i_* > 2*c*_B;_ these bacteria are shown as white dots in Fig. 1B). The proportion of strongly accumulated cells was maximal (37.4 ± 11.7%) at the highest patchiness (i.e., at lowest background, *c*_B_ = 0.4 μM, obtained with Δ*t* = 105 s) and consistently decreased (to 36.4 ± 8.5%, 21.0 ± 5.5%, and 9.2 ± 4.7%) with decreasing patchiness, as the background in the different experimental treatments increased (to *c*_B_ = 1.2, 3.6, and 8.1 μM, respectively). This effect is also apparent from the distribution of the potential uptake rescaled by the background concentration (Fig. 1C, inset).

### Bacterial clustering follows predictions from sensory noise theory

The observation that a higher proportion of cells accumulated around nutrient patches in landscapes with sparser hotspots can be understood theoretically by mapping the bacterial arena according to the signal-to-noise ratio SNR = $\left|\nabla c\right|$ *v*/σ_c_ associated with bacterial chemotaxis [by plotting regions in which SNR(*x*,*y*) > 1; Fig. 1D]. For a bacterium performing temporal gradient sensing, the magnitude of the rate of change in concentration experienced is the product of the magnitude of the gradient, $\left|\nabla c\right|$, and the cell’s swimming speed, *v*. To reliably climb a gradient, the cell’s measurement of the signal must exceed the sensory noise, σ_c_, a critical parameter that limits the regions in which bacteria can detect and climb chemical gradients (*26*, *31*). The noise arising from the stochasticity of the encounters that the swimming cell has with chemoattractant molecules is proportional to the square root of the local chemical concentration, σ_c_ ∝ *c*(*x*,*y*,*t*)^1/2^ (Materials and Methods) (*32*, *33*). Therefore, where and when cells can climb a gradient depends not only on the steepness of the gradient but also on the magnitude of the concentration. As the nutrient landscape becomes more complex, as in our experiments, a framework is needed that incorporates both signal and noise to determine when and where cells can climb gradients to navigate toward nutrient hotspots.

For higher values of the background chemical concentration *c*_B_, the noise associated with gradient measurements may hinder detection of a chemical patch with concentration *c*_P_(*x*,*y*,*t*). For a swimming *V. anguillarum* cell, this manifests in (i) a maximal radius r* from the patch, beyond which gradients cannot be detected, and (ii) a patch persistence time t*, after which gradients cannot be detected. Because the overall concentration experienced by a bacterium is the sum of the pulse and background combined, i.e., *c*_P_(*x*,*y*,*t*) + *c*_B_, the molecular counting noise scales as σ_c_ ∝ (*c*_P_(*x*,*y*,*t*) + *c*_B_)^1/2^. The values r* and t* represent the radial distance from the center of a patch and time after its release, respectively, where the SNR drops below 1. Henceforth, we use these values to quantify (i) the maximum spatial range of chemotactic interaction of bacteria with a nutrient pulse, and (ii) the total lifetime of the interaction, from the pulse creation via LED activation to its dissipation.

Building on this theory, we developed a method to derive the spatial and temporal extent (r*, t*) of patches associated with chemical pulses spreading in backgrounds of different magnitude. We found that patch extent follows two distinct scaling regimes: At negligible background concentrations, the patch lifetime and extent scale as t* ∝ *M*^1/2^*v/*χ and r* = (12Dt*)^1/2^, respectively, with the factor χ related to a bacterium’s chemotactic capability (lower value of χ corresponds to greater chemotactic precision; see Supplementary Methods). These values depend on the bacterial sensory properties, allowing calculation of the spatial and temporal extent where bacteria can sense the patch. In contrast, for high background concentrations, these values scale as t* ∝ *M*^1/3^(*v/*χ)^2/3^ and r* = (8Dt*)^1/2^, respectively (figs. S3 and S4). For pulses of mass M* = 2.3 × 10^9^ μm^−1^ as in our experiments, the radius r* ranges from a few hundred micrometers (r* = 498 μm) for pulses released at the highest background (*c*_B_ = 8.1 μM) to just over 1 mm (r* = 1137 μm) for those released at the lowest background (*c*_B_ = 0.4 μM), with the pulse lifetime t* ranging from 89 to 204 s, respectively. In other words, the ability of a nutrient patch to elicit a chemotactic response depends not only on the patch’s physical size, but on the background concentration of nutrients, which is in turn determined by the number of other patches in the system. To investigate how bacterial foraging plays out in the ocean, it is essential to replicate an arena that is sufficiently large to study all relevant regimes of patch density. Owing to the large spatial extent of patches in low backgrounds, the requirement of widely separated patches demands arenas much larger than r*, a condition that until now has proven experimentally elusive and that our experimental arena (*L* = 10 mm) satisfies.

The number of patches active at any given time (Fig. 1D), obtained by calculating the ratio *N* = t*/Δ*t* of the pulse lifetime over the time interval Δ*t* between the release of two consecutive pulses, demonstrates that, on average, there are multiple patches detectable by bacteria in the population across the domain at any instant in time (average *N* ≈ 2 to 20 in the 1-cm^2^ domain). The instantaneous patch density can be calculated as ρ = *Ν*/*Α*, resulting in an average interpatch distance of *r*_min_ = (2πρ)^−1/2^. The accumulation dynamics of bacteria are governed by the extent to which patches are well separated in space and time, as determined by the relative magnitudes of r* and *r*_min_. In the case where r* < *r*_min_/2, patches are widely separated, and the length scale relevant for bacterial accumulation is r*. Conversely, for r* > *r*_min_/2, patches are dense and more frequently overlapping, hence the length scale relevant for accumulation is *r*_min_/2. As a single reference scale across the two regimes, we therefore define the characteristic nutrient length scale, *r*_C_, as the smaller of half the interpatch distance *r*_min_/2 and the patch size r* predicted by theory (Fig. 2B), i.e., *r*_C_ = min(*r*_min_/2, r*).

**Fig. 2.**
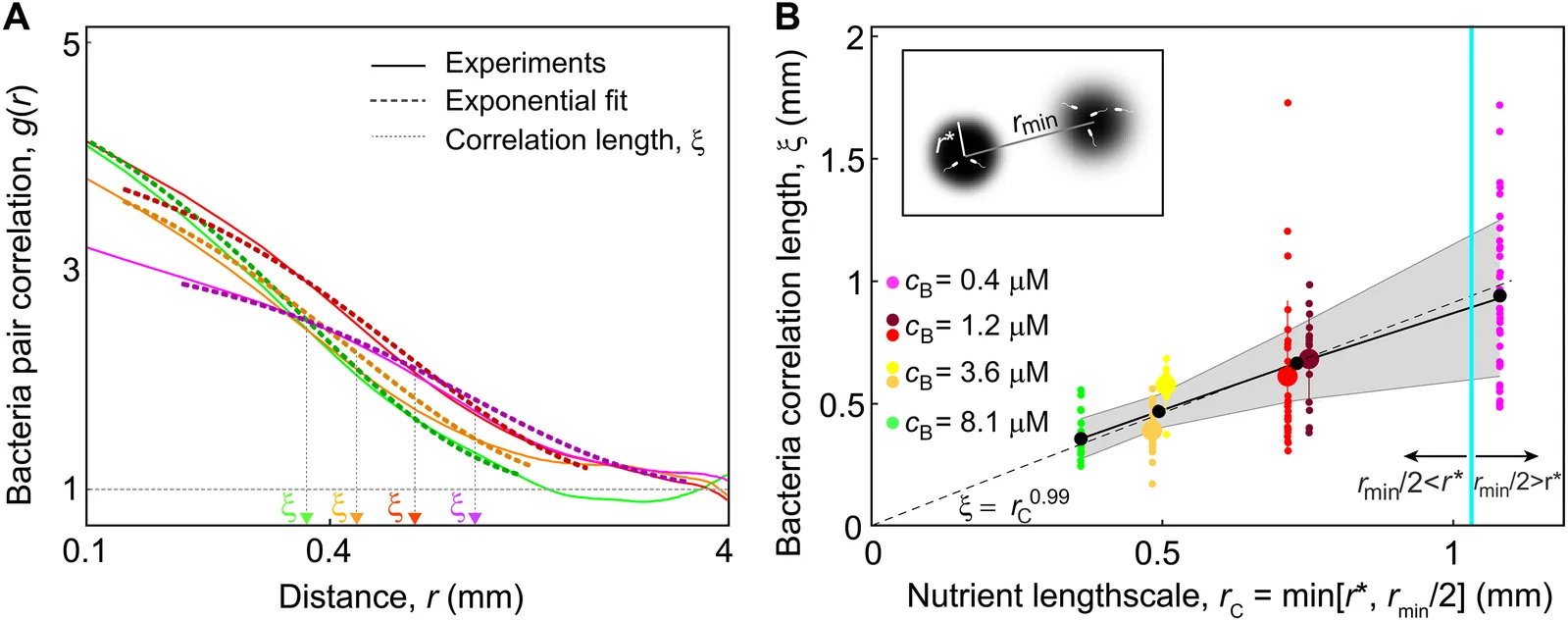
Bacterial accumulation scales with the size of nutrient patches. (**A**) Bacterial pair correlation function, *g*(*r*), on semilog scale, as a function of the distance *r* between two bacterial cells. This represents the probability of finding bacteria at a distance *r* from a given reference bacterium, relative to that for a random distribution. Colors [see (B) for color legend] represent different levels of patchiness, equivalent to changes in the background concentration *c*_B_. Dashed lines show exponential fits of the form e^−*r*/ξ^, with the correlation length ξ representing the characteristic size of bacterial accumulation (see fig. S5 for all experimental replicates). (**B**) The bacterial correlation length ξ as a function of the nutrient length scale *r*_C_ shows a linear scaling (black dashed line, best-fit exponent: 0.99 ± 0.02). The nutrient length scale is the characteristic size of the landscape perceived by a chemotactic bacterium and is computed as the smaller between half the interpatch distance *r*_min_ and r* (see inset); r* is the maximum radial size of a pulse, defined as the region where the SNR > 1 (fig. S3). The vertical cyan line indicates the radial distance at which r* > *r*_min_/2 in our experiments. Larger colored circles represent the different replicates, and small dots represent different time points within each replicate.

The tightness of bacterial clustering decreases with the characteristic nutrient length scale *r*_C_. We characterized the bacterial clustering tightness around individual hotspots by a length scale, ξ, extracted by fitting an exponential curve, exp(−*r*/ξ), to the pair correlation function *g* for each nutrient landscape (Fig. 2A and fig. S5). The pair correlation *g*(*r*,*t*) measures the probability, at time *t*, of finding bacteria at a distance *r* from a given reference bacterium, relative to that of a random distribution: A uniform distribution of bacteria would result in *g* = 1. The statistical properties of patch release do not change over time (i.e., the process is stationary), so we computed the time average, *g*(*r*), given the bacterial positions acquired at all times *t*. Across all experiments, results revealed that for *r* < 100 μm, one has 3 < *g*(*r*) < 6 (fig. S5), meaning that within 100 μm of a patch center, bacteria were, on average, three- to sixfold more concentrated than in the far field.

### Foraging efficiency exhibits two scaling regimes

The bacterial sensing probability, computed as the total fraction *f*_A_ of the arena where bacteria can perform chemotaxis toward chemical pulses (SNR > 1), shows two distinct scaling regimes depending on the patch density. At low patch density, the sensing probability *f*_A_ scales linearly with increasing nutrient flux ϕ (and background *c*_B_) before saturating to a sublinear scaling regime where *f*_A_ ∝ ϕ^1/3^ (Fig. 3A, magenta and green curves). We computed the sensing probability as *f*_A_ = 1 − exp[−πr*^2^t*/(Δ*tA*)], which can be approximated at low density by the expression *f*_A_ ≈ πr*^2^t*/(Δ*tA*). By inserting the scaling relations for r* and t* at low background concentration into the expression for *f*_A_ and recalling that *c*_B_ = M*/(*A*Δ*tN*_A_), we find the linear scaling *f*_A_ ∼ ϕ between sensing probability and nutrient flux (or background). We call this the “linear sensing regime.” At high patch density (and therefore high background), the sublinear scaling *f*_A_ ∼ ϕ^1/3^ is obtained through a similar argument (see the Supplementary Materials)—we call this the background-dominated sensing regime.

**Fig. 3.**
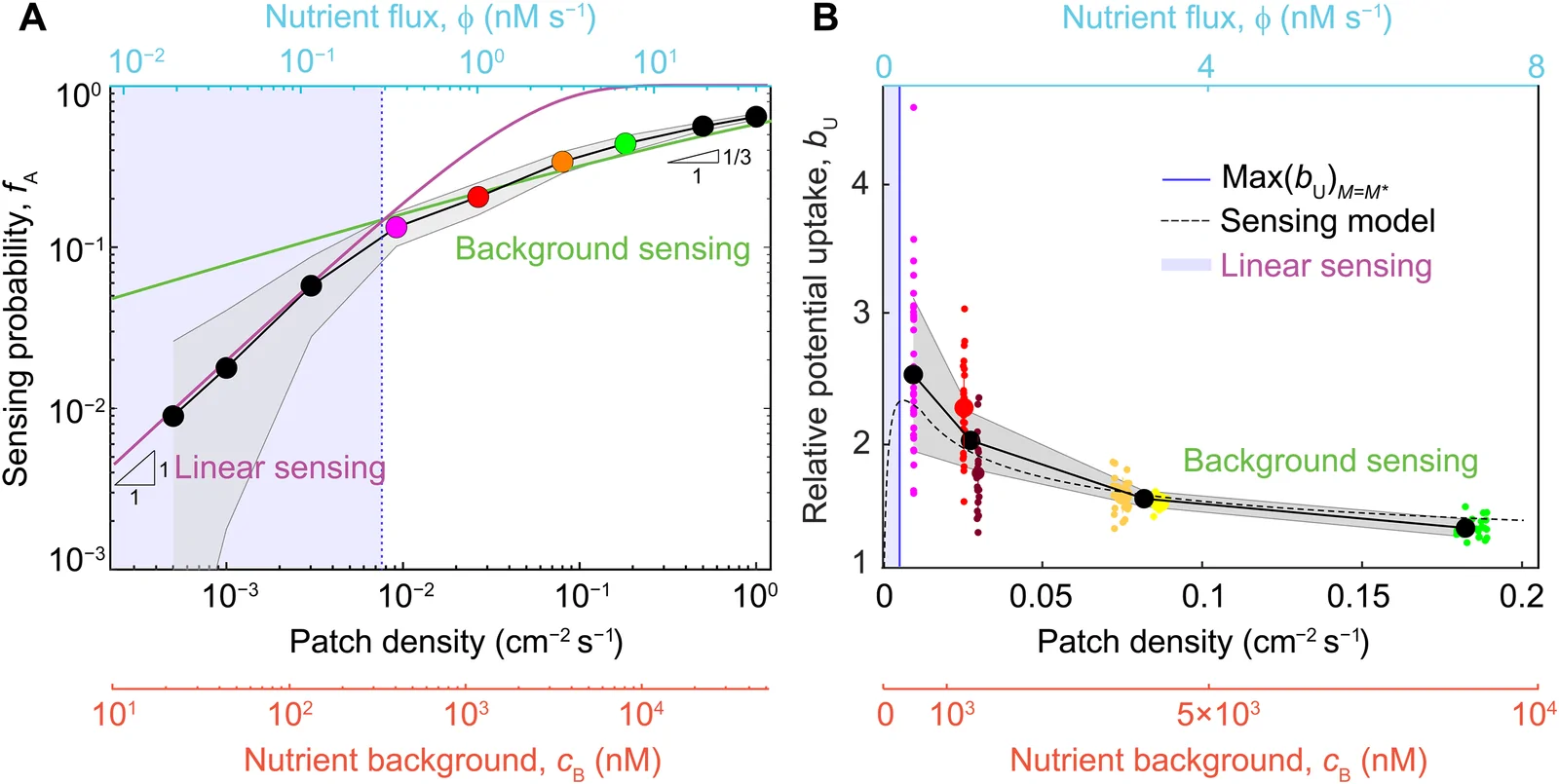
Bacterial sensing and foraging efficiency are controlled by microscale patchiness. (**A**) The sensing probability *f*_A_ as a function of the patch density. The sensing probability is the average fraction of the experimental area where bacteria can actively climb gradients [that is, where SNR(*x*,*y*) > 1]. Circles represent *f*_A_ values obtained using the SNR from nutrient profiles at a given patch density (colored circles correspond to experimental landscapes, black circles represent numerical simulations outside the experimental range). The additional *x* axes provide the conversion from patch density to values of nutrient flux ϕ (cyan *x* axis, above the plot) and nutrient background *c*_B_ (red *x* axis, below the plot) obtained by using a washing timescale τ in the experimental arena (Materials and Methods). Magenta and green lines represent the predictions from sensory noise theory for linear sensing, applicable at low patch densities (“open ocean” conditions), and background sensing (with scaling exponent α = 1/3), applicable at higher patch densities (“bloom” conditions), respectively (see the Supplementary Materials). (**B**) The experimentally determined potential uptake (i.e., nutrient exposure) of cells relative to a nonchemotactic population, *b*_U_, decreased with increasing patch density, in agreement with the prediction from the sensing model (dashed black line; see the Supplementary Materials). Decreasing patch density corresponds to the transition between background sensing and linear sensing. This transition occurs where *b*_U_ is maximal, as highlighted by the blue vertical line. Larger colored circles represent the different replicates (mean ± SD), and small dots represent different time points within each replicate. The color code corresponds to the same parameters as in Fig. 1. Black circles are the mean values for all experiments at a given patch density, with the shaded area representing ±1 SD.

The transition between these two scaling regimes determines the patch density at which the foraging efficiency *b*_U_—computed as the nutrient uptake, *u*, for a bacterial population over the background *c*_B_—is maximum. Beyond this patch density, gradient climbing is impaired by a higher background and there is diminishing uptake return if patch density increases further. In the background-dominated sensing regime, gradient climbing, and therefore population uptake, is impaired by an excessively high nutrient background relative to the patch strength *M*. Conversely, in the linear sensing regime, patches occur too rarely in space and time to boost the foraging efficiency overall. Although the effective radius of the region where bacteria can chemotax to a pulse is larger in this sparse regime, there are so few pulses that only a small fraction of the population is exposed to them. Thus, the effect of chemotaxis to nutrient pulses is limited by the fact that very few bacteria experience a pulse.

The patch prominence (ε_Μ_), defined as the ratio of nutrient concentration *c*_P_ from the pulse at (r*, t*) over the background concentration, i.e., ε_Μ_ = *c*_P_(r*,t*)/*c*_B_ (Fig. 4A), follows the *c*_B_ ∝ *M*^1/2^ scaling imposed by the SNR (eq. S11; Supplementary Methods). The transition between the two scaling regimes occurs at values of patch prominence ε_Μ_ ∼ 1, as a direct consequence of its definition. The background-dominated and the linear sensing regimes pertain to the parameter space for which *c*_P_ (r*, t*) << *c*_B_ and *c*_P_ (r*, t*) >> *c*_B_, respectively (see the “Sensing probability” section in the Supplementary Materials).

**Fig. 4.**
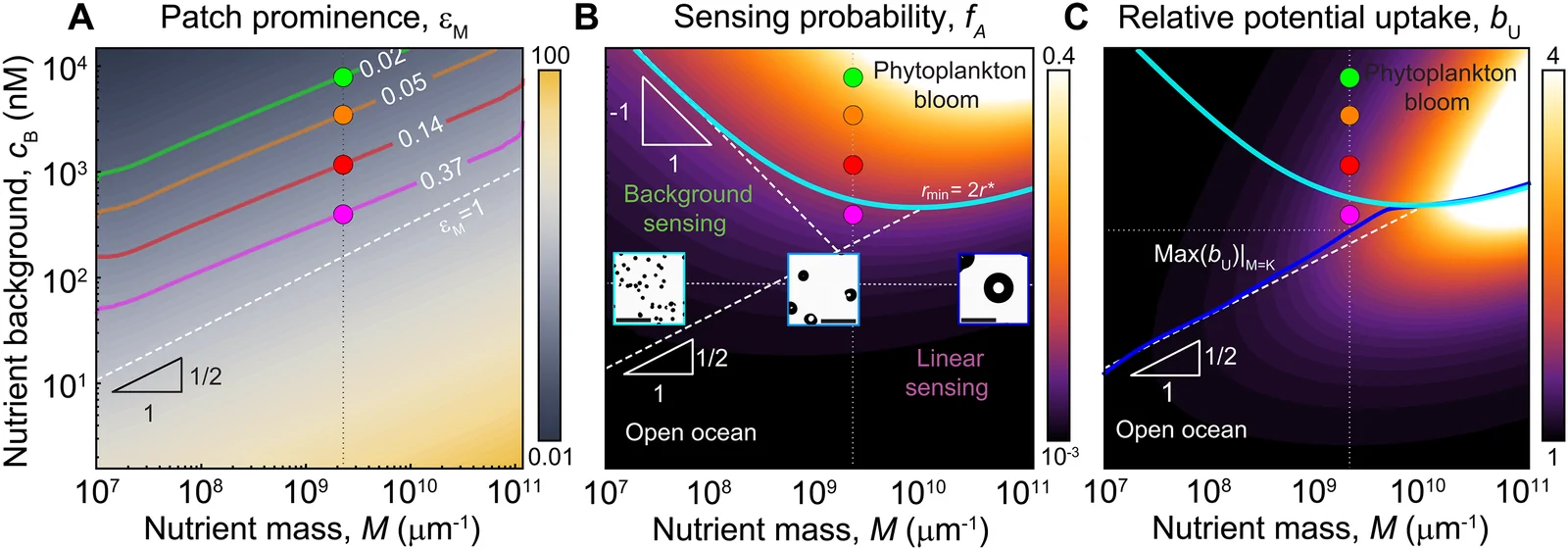
Nutrient patchiness controls bacterial foraging efficiency. (**A**) Patch prominence, ε_M_, defined as the ratio between patch nutrient concentration and background concentration at time t* and radius r*. Results are shown as a function of the nutrient mass *M* within a pulse, and the nutrient background *c*_B_. Isoclines of same prominence (colored curves, with ε_M_ = 1 as a white dashed line with scale exponent $c_{B}\propto M^{1/2}$). Colored circles represent the experimental conditions, characterized by different background concentrations *c*_B_ and the same pulse mass, M* = 2.3 × 10^9^ μm^−1^. (**B**) Sensing probability, *f*_A_, as a function of *M* and *c*_B_. The cyan curve delimits the region (above the curve) where nutrient patches start to overlap in space (*r*_min_ = 2r*). The white dashed lines (with slope 1/2 and −1) show the transition between the linear and background sensing regimes. The three insets show representative SNR maps (black regions correspond to SNR > 1; see Fig. 1) from nutrient regimes having the same background $c_{B}$ but different masses *M* (compare with Fig. 1D where SNR maps are shown for constant *M* and different *c*_B_). Colored circles as in (A). (**C**) For environments of a given nutrient mass *M*, the value of $c_{B}$ corresponding to the maximum relative uptake *b*_U_ (dark blue curve) coincides closely with the transition between linear sensing and background sensing regimes (see agreement between blue curve and white dotted line). *b*_U_ is computed by rescaling the potential nutrient uptake of the population with that of a population of randomly distributed cells. Colored circles as in (A).

Experimental values of relative nutrient exposure, computed by superimposing the measured bacterial distribution on the reconstructed chemical landscape, follow predictions from sensory noise theory over the four patch densities tested (Fig. 3B). To quantify the potential uptake of a chemotactic population foraging in a patchy landscape, we multiply the sensing probability *f*_A_ (the proportion of the domain where chemotaxis is possible) by the uptake benefit *B* gained from a patch (time-integrated nutrient concentration from a single patch; see the Supplementary Materials). We also numerically estimate the uptake for uniformly distributed bacteria, so as to have a baseline uptake for nonchemotactic cells in the same landscape. The ratio of these terms, which is equivalent to the foraging efficiency, quantifies the relative enhancement in uptake due to chemotaxis. Results for potential uptake relative to an in silico nonmotile population are similar, with the position of the maximum slightly shifted toward higher values of patch density because of the energy expenditure from swimming (fig. S6).

Patchiness can be modulated by tuning both the number and the strength of the nutrient pulses, mimicking the appearance in the coastal ocean of different nutrient sources, ranging, for example, from small, abundant cyanobacteria (*30*) to large, rare diatoms (*34*). By applying sensory noise theory over a broader region of parameter space than that explored experimentally (Fig. 4, A and B), we computed the sensing probability and the uptake efficiency of populations foraging over a broad range of ecologically relevant landscapes. For example, it is possible to fix the flux of nutrients injected over the arena ϕ (i.e., same background *c*_B_) but create differing spatiotemporal distributions by simultaneously changing the intensity *M* and frequency Δ*t*^−1^ of the pulses while keeping their ratio constant (Fig. 4B, inset; see the three SNR maps). This analysis revealed that high-intensity, yet sparse, pulses are associated with an up to fourfold higher foraging efficiency compared to low-intensity, yet frequent, pulses (Fig. 4C). At the same time, landscapes with higher patchiness also showed higher uptake inequality among cells at any given time, as captured by the Gini index (*35*) (fig. S8; Supplementary Methods).

### Long-term plasticity of bacterial foraging

By further analyzing the experimental trajectories of individual bacteria over space and time, we found that faster cells were associated with a higher potential nutrient uptake (Fig. 5A). This association increased over time during the experiments and was stronger for stronger patchiness (fig. S7): The slope of a linear fit of swimming speed as a function of potential uptake was highest (1.38 ± 0.02 μm s^−1^ μM^−1^) at the lowest patch density (i.e., the highest patchiness, *c*_B_ = 0.4 μM), increasing by 72% from 1.00 ± 0.03 μm s^−1^ μM^−1^ at the beginning of the experiment to 1.72 ± 0.05 μm s^−1^ μM^−1^ at the end after several hours of treatment (see fig. S7 for full results). For the lowest patchiness (i.e., the highest patch density, corresponding to *c*_B_ = 8.1 μM), the slope of the linear fit reduced to 0.21 ± 0.01 μm s^−1^ μM^−1^. A cell’s ability to sense a chemical gradient, and therefore its sensing probability, *f*_A_, within the domain depends on its swimming speed *v*, because the cell measures the ramp rate ∇*c*·**v** to bias its motion up the gradient (*15*) and because swimming speed dictates the rate at which the cell climbs the gradient once it has sensed it (*36*).

**Fig. 5.**
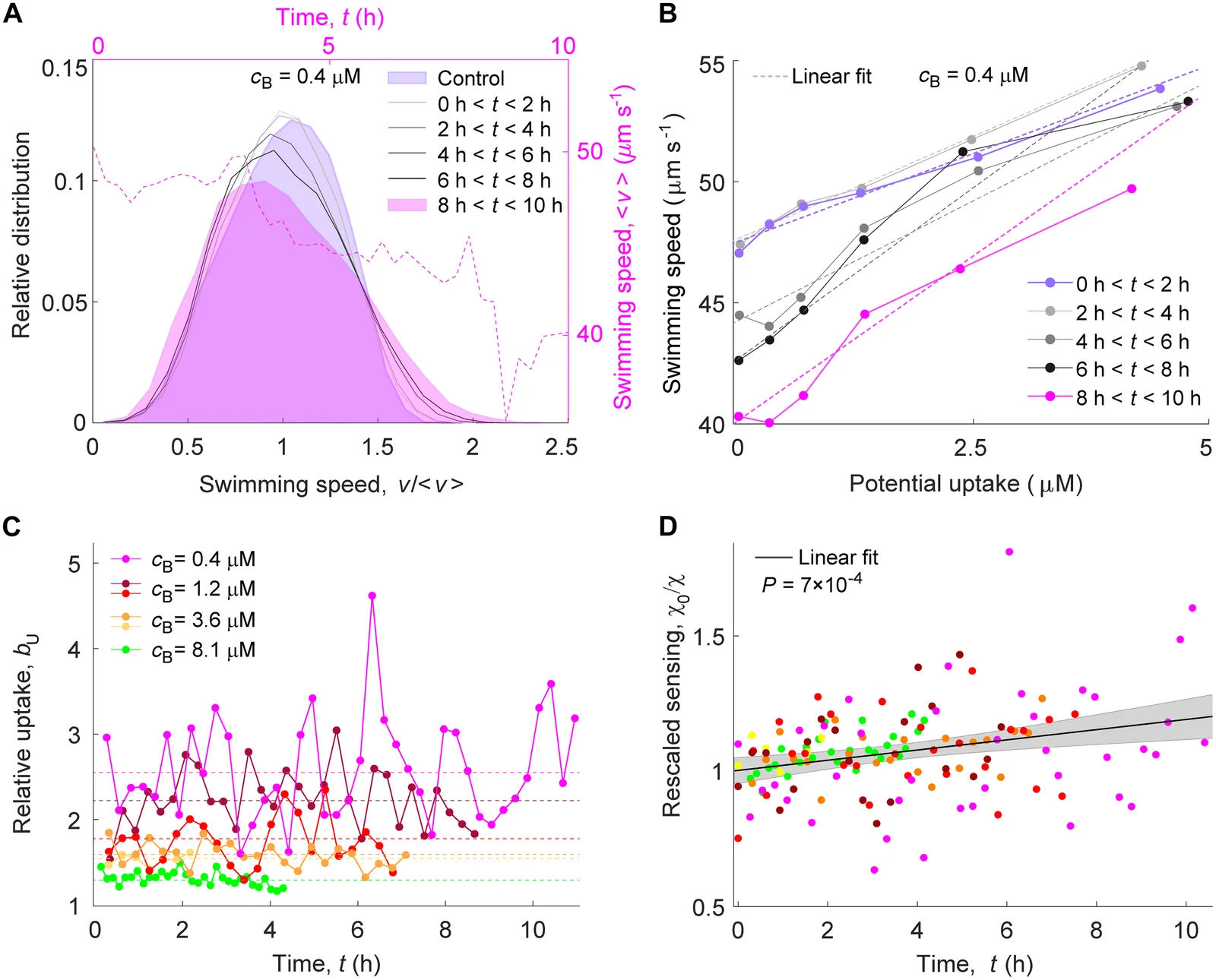
Temporal evolution of swimming speed and potential nutrient uptake shows long-term plasticity of chemotactic performance in *V. anguillarum*. (**A**) Relative distribution of swimming speed *v* across a population, rescaled by the mean speed <*v*>, for *c*_B_ = 0.4 μM (see fig. S7 for experimental results for all values of *c*_B_). The distribution broadens with time (see legend). The mean swimming speed decreases by ∼20% (from 50.3 to 41.1 μm s^−1^) over 10 hours of experimental treatment (dashed line and magenta *x* and *y* axes). (**B**) The swimming speed increases with the potential uptake as shown by a positive slope. Dashed lines are linear fits, and circles are experimental data at nutrient background *c*_B_ = 0.4 μM (see fig. S7 for experimental results for all values of *c*_B_). Colors correspond to different time windows (legend). (**C**) The relative potential uptake, *b*_U_, as a function of time fluctuates around an average value (dashed lines), for all nutrient backgrounds *c*_B_ (color-coding, see legend), with a variance that depends on *c*_B_. (**D**) The chemotactic precision χ^−1^ (see Supplementary Methods) rescaled by its value at time zero, χ_0_, for different nutrient backgrounds *c*_B_ [colors; see legend in (C)] increases over time. The black line and shaded region show the best linear fit and 95% confidence interval and the legend gives the *P* value from Pearson’s correlation. h, hours.

Swimming speed declined by about 20% from 50.3 to 41.1 μm s^−1^ over the course of the experiments (Fig. 5B and fig. S7). This was accompanied by a broadening in the distribution of swimming speeds across the population, as shown by the coefficient of variation of the speed distribution increasing from CV_v_ = 0.28 at the beginning to CV_v_ = 0.37 at the end of the experiment, at the highest patchiness (Fig. 5A; see fig. S7 for full results). However, the potential uptake remained, on average, constant over time (Fig. 5C), with fluctuations reflecting those naturally present in the respective nutrient landscape (fig. S9). This indicates that the chemotactic precision of *V. anguillarum* increased throughout the experiment (Fig. 5D). The fact that timescales over which bacteria module their chemotactic precision can be carefully characterized in varying dynamic environments could provide clues about the mechanisms involved in behavioral plasticity of foraging bacteria.

## DISCUSSION

At the microscale, the ocean is a patchwork of ephemeral nutrient hotspots interspersed within a nutrient-poor water column. Nutrient patches arise from diverse sources spanning several scales, from cyanobacterial exudates or lysing phytoplankton (few micrometers) (*7*, *37*, *38*), through to zooplankton excretions or the plumes (*39*) diffusing behind sinking marine snow (hundreds of micrometers to centimeters). The ability of copiotrophic bacteria to locate and exploit these transient nutrient patches through chemotaxis is central for their survival and contributes to set the balance between nutrient remineralization and carbon sequestration to the deep ocean in marine ecosystems (*10*, *11*). However, experimentally reproducing the range of ecologically relevant conditions in a laboratory system that also allows one to study how bacteria respond behaviorally to dynamic patchiness has not been feasible in the past.

Here, using the marine bacterium *V. anguillarum* chemotaxing toward the amino acid glutamate as a model system, we developed a method to study long-term bacterial foraging under microscale patchiness in a microfluidic device capable of reproducing the types of nutrient landscapes that bacteria encounter in the wild, through the sequential creation of microscale chemical pulses. One of the major experimental impediments in chemotaxis research, but also more generally in ecology, has been the difficulty of experimentally creating complex, dynamic resource environments with multiple sources that are in a statistical steady state, i.e., not just a steady gradient or a single spreading hotspot, and without a progressive buildup of background concentration. In our device, the nutrient flux at the microscale is balanced by a homogeneous washing rate, achieving statistical steady state conditions, allowing us to isolate variables like patchiness and background concentration. By tuning the balance between nutrient flux and washing rate, we were able to impose patchiness corresponding to a broad range of productivities typical of coastal seawaters, where background DOM concentrations are in the range of hundreds of nanomolar [representative of oligotrophic conditions; (*29*)] to low micromolar [occurring for example during phytoplankton blooms; (*34*)]. A second innovation of our system is the ability to simultaneously quantify population-scale responses to dynamic resources, and individual-level behavioral responses at a much finer scale. This ability to connect behavioral strategies of individual cells with population-scale movements and performance allowed us to study how bacterial foraging strategies perform across a range of conditions engineered to closely mimic the ones these organisms experience in nature. Specifically, by using the observed bacterial aggregation in response to microscale patchiness over several hours and the predicted chemoattractant landscape over that same time frame, we quantified the potential nutrient uptake of many individual bacterial cells distributed across the environment. Combining this with estimates of spatial cell density allowed us to estimate population uptake. We further leveraged the high-resolution tracking of cells over timescales of seconds to find long-term changes in bacterial chemotactic motion under naturally relevant nutrient regimes.

Marine bacteria foraging in the ocean are subject to trade-offs between resource acquisition and the energetic cost of swimming (*40*), including the maintenance of the chemotactic machinery (*41*, *42*). Bacterial swimming, both directed for chemotactic motion or diffusive in the absence of detected signals, may increase the rate of nutrient acquisition by enhancing the encounter rate with resources. However, swimming also increases the energetic demands on the cell, especially during the search for resources when cells may not have access to nutrients and their physiology is adapted to starvation conditions (*43*). Our experimental results showed that in populations foraging in nutrient landscapes mimicking marine environments, a progressive reduction of the average swimming speed was compensated for by a sharpening of the chemotactic precision, so that the cells maintained a stable nutrient exposure over time. This may represent a strategy by which bacteria could reduce the costs of swimming without compromising their access to resources under low nutrient conditions, although including the energetic cost of maintaining higher chemotactic precision may partially offset this.

Furthermore, the broadening of the swimming speed distribution over time (Fig. 5A and fig. S7) suggests that underlying positive feedback mechanisms between bacterial traits, such as swimming speed or chemotactic precision, and nutrient exposure under microscale patchiness could further increase the naturally occurring population heterogeneity in bacterial traits (*16*, *44*, *45*). Investigating realistic ecological-physiological feedback mechanisms involving cell behavior, nutrient uptake, and the chemical microenvironment is afforded here through long-term imaging of dynamic pulses in a statistical steady state. Achieving long-term, single-cell tracking of highly diluted populations of bacterial cells foraging under microscale patchiness, possibly accompanied by transcriptomic and proteomic analyses, would further elucidate these feedback loops and their roles in the foraging and physiological changes we observed. A future characterization of the energetic trade-offs involved in behavioral plasticity should also include the cost associated with adjusting the chemotactic precision.

Bacterial chemotaxis is constrained by the limits of the sensory noise associated with the stochastic binding-unbinding events of chemoattractant molecules to the chemoreceptors (*26*, *33*) as well as internal noise in signal processing and behavioral responses (*46*). The environmental signal most relevant to chemotaxis is thus spatiotemporal variation of the gradient SNR and not simply the gradient. For bacteria that forage in patchy environments, SNR strongly influences when and where bacteria can detect and respond to chemical gradients and accumulate around nutrient sources (*27*). Here, we applied sensory noise theory to the case of chemical pulses diffusing in a quiescent fluid to model our experimental conditions. However, both the SNR formalism and the experimental platform are applicable across other species and chemoattractants and can be extended to diverse environmental conditions, such as leaky nutrient sources (with steady as opposed to unsteady chemical profiles) and the presence of fluid flow, or to diverse ecological settings, such as multiple species to investigate community dynamics. More generally, the partitioning of complex nutrient landscapes into zones of active chemotaxis enabled by the SNR formalism provides a powerful tool to investigate how bacterial foraging efficiency is affected by microscale nutrient patchiness and could represent a foundation for including microscale nutrient uptake dynamics in mesoscale ecosystem models. Beyond bacterial chemotaxis, this work exemplifies the ecological importance of spatiotemporal heterogeneity and dynamic behavioral responses to it. This reveals how individual-scale decision-making scales up to influence population-level uptake, in an experimental device crafted to reproduce realistic spatiotemporal statistics of the ecological environments experienced by organisms in the wild.

## MATERIALS AND METHODS

### Bacterial isolates and cell culture

For the multiple chemical-pulse experiments presented in the main text, we used GFP-tagged *V. anguillarum* 12B09 chasing chemical pulses of the amino acid glutamate. For the isolate harboring pGFP, spectinomycin (50 μg/ml) was added to maintain the plasmid. Cultures of *V. anguillarum* 12B09 were grown in 1/2 strength 2216 Marine Broth (BD Difco, Thermo Fisher Scientific) at 30°C on an orbital shaker (600 rpm) for 20 hours, and cells were harvested in late-exponential phase (OD_600_ ∼ 1, which, for *Vibrios*, typically corresponds to ∼10^9^ cells ml^−1^). Cells of *V. anguillarum* were washed by centrifuging 1 ml of cell suspension at 2500*g* for 3 min, removing the supernatant, and gently resuspending in 1 ml of filtered autoclaved artificial seawater (Instant Ocean, Aquarium Systems Inc.) at salinity 36 g kg^−1^, using a micropipette to avoid flagellar damage by mechanical shearing forces. The population of *V. anguillarum* was then starved on an orbital shaker (600 rpm) at 30°C for 3 hours.

### Fabrication of the 3D-printed microfluidic device

The 3D shape of the large bacterial arena was designed using 3D-design software, and the master for the polydimethylsiloxane (PDMS) mold was printed with a high-resolution 3D printer (fig. S1B) (*23*). The fabrication of the molds was performed using a commercial 3D-printing service. However, similar results can be achieved using a 3D printer, with a minimal resolution of 50 μm to resolve the smallest features of the microchannel designs, provided that the 3D-printed material is sufficiently smooth to achieve a good bonding between the cast PDMS (Sylgard 184, Silicone Elastomer Kit, Dow Corning) and the other surfaces of the device (i.e., glass, polystyrene, and PDMS). The 3D PDMS was bonded to a petri dish (60 mm by 25 mm), after activating both the surface of the petri dish and the PDMS with oxygen plasma (Diener electronic, Zepto B model 1 plasma oven) for 1 min. The petri dish was bonded to the PDMS in an oven at 45°C for at least 12 hours to strengthen the chemical bonds. To activate the nanoporous polycarbonate (PCTE) membrane (Whatman Nuclepore Track-Etched; pore size, 0.4 μm; diameter, 47 mm), both surfaces of the membrane were exposed to oxygen plasma for 1 min. Under a chemical hood, a commercial solution of 3-aminopropyltriethoxysilane (APTES, A3648 Sigma-Aldrich) was diluted in deionized water to 0.1% by volume, and the activated membrane was immersed in the APTES solution for 20 min using tweezers. The membrane was then placed on a cleanroom wipe to dry under the chemical hood flow for at least 1 hour. The PDMS washing microchannels were bonded to the functionalized membrane after activation with oxygen plasma chamber for 2 min by bringing the PDMS washing channel and the functionalized membrane into contact and gently pressing the PDMS washing channel. The membrane was then sandwiched between the two PDMS layers (fig. S1D) after activation of both the bonded PDMS washing channel–PCTE membrane and the 3D PDMS mold–petri dish with an oxygen plasma chamber for 2 min. The device was finally placed in an oven at 45°C for at least 12 hours to strengthen the chemical bonds.

### Preparing the microfluidic chip with cells and caged chemoattractant

Prior to chemotaxis experiments, the microfluidic chamber was maintained under vacuum for 20 min to minimize the occurrence of air bubbles in the bacterial suspension during filling. The microfluidic chamber was extracted from the vacuum pump and the bacterial arena below the nanoporous polycarbonate (PCTE) membrane was immediately filled with a diluted bacterial suspension of GFP-fluorescent *V. anguillarum* cells. Cells from the starved culture were diluted for use in experiments to a final concentration of 1 × 10^7^ cells ml^−1^ to allow the tracking of individual cells at low magnification (4× objective). To the cell suspensions, 4-methoxy-7-nitroindolinyl-caged-l-glutamate (MNI-caged-l-glutamate, catalog no. 1490, Tocris Bioscience) was added. These chemical modifications of the amino acids by a photoremovable protecting group render the molecules biologically inert and remove any chemoeffector activity of the amino acids.

Prior to the experiments, a stock artificial seawater solution of 10 mM MNI-caged-l-glutamate was prepared and stored at −20°C in the dark to protect them from light, thereby preventing photolysis. On the day of the experiment, the stock solution was diluted in artificial seawater (1 mM MNI-caged-l-glutamate solution) to fill a 20-ml syringe attached by tubing to the inlet of the washing channel above the PCTE membrane (fig. S1). The tubing was then connected to a waste reservoir, entirely submerged in the fluid waste to avoid pressure oscillations. The solution of 1 mM MNI-caged-l-glutamate was injected at constant flow rate (*Q* = 50 μl min^−1^) using a syringe pump (Harvard Apparatus PHD) to obtain the desired mean flow velocity (*u* = 200 μm s^−1^) in the washing channel (width, 2 cm; height, 200 μm; fig. S1).

### Uncaging procedure

The molecules of MNI-caged-l-glutamate are uniformly present in the bacterial suspension but cannot be detected by the cells until their sudden release, which occurs at user-defined points in time and space by exposing the arena to a near–UV-A (395 nm) focused LED beam (SPECTRA X light engine, Lumencor). Custom-made software controlling the LED beam and microscope stage generated user-defined spatiotemporal sequences of pulses {*p*_1_,…,*p_n_*} at different random locations {(*x*_1_,*y*_1_,*t*_1_),…,(*x_n_*,*y_n_*,*t_n_*)} in the bacterial arena and interspaced with a fixed interarrival time, *Δt*, which was varied in the range 5 to 105 s to modulate the patchiness. The release of the nutrients occurred over a square of area *A* = 1 cm^2^ located in the center of the bacterial arena, whose total extension is 4 cm^2^ (fig. S1C). This procedure ensures the implementation of leaky boundary conditions over the perimeter of *A* and avoids edge effects at the physical boundaries of the bacteria arena. By tuning the LED beam exposure time (in our case, *t*_P_ = 500 ms), pulses of prescribed initial concentration were released (in the experiments, *c*_0_ = 35 μM) over the specific location illuminated by the beam (radius of LED pulse, *r*_P_ = 175 μm). By modulating *c*_0_ or Δ*t*, it is possible to manipulate the flux of nutrient, ϕ, and therefore the background, *c*_B_, in both its mean value and standard deviation (for example, by keeping constant the ratio *c*_0_/Δ*t*). The number of molecules released in the pulse is determined by a calibration relationship between exposure time and uncaging fraction (*23*, *26*), where the absorption spectrum after photolysis is characterized using UV-Vis spectroscopy (Nanodrop One UV-Vis Spectrophotometer, Thermo Fisher Scientific).

### Imaging procedure

The petri dish containing the microfluidic chip was placed on an inverted microscope (Nikon Eclipse Ti-E), and cells were imaged in fluorescent light using the LED beam (SPECTRA X) implemented for the photolysis of the amino acid molecules, but activating a different light channel at a wavelength λ = 488 nm. The capacity for phase contrast imaging is impaired because of the presence of the PCTE membrane in our device. We used a 4× objective lens (NA, 0.13; estimated depth of field, *d* = 37 μm) to visualize bacterial dynamics, focusing at midchamber (*z*_F_ = 250 μm). Video imaging was conducted at regular time intervals (*T*_D_ = 15 to 30 min) over 3 × 3 contiguous locations (following a snake pattern) for a short period of time (*T*_I_ = 1.25 s) in each location. This allowed us to perform bacterial tracking at each time increment *T*_D_. In this way, we could reconstruct the bacterial positions and swimming speed over the large square surface *A* = 1 cm by 1 cm of the bacterial arena, by stitching together the nine tiles (each with a field of view of 3.34 mm by 3.34 mm) recorded at each time increment *T*_D_. Videos were captured at a frame rate of 12 fps with an sCMOS camera (Andor Zyla 5.5) controlled through Nikon Elements software. We reconstructed bacterial trajectories with custom in-house MATLAB (MathWorks) scripts. From these trajectories, we calculated position and swimming speed for each bacterium at each time increment. From the reconstructed bacterial trajectories, we could determine the spatiotemporal dynamics of the distribution of bacteria over the whole surface *A* = 1 cm^2^ as they respond to the microscale chemical pulses (Fig. 1B). Each experiment lasted a period of several hours (*T*_E_ = 5 to 10 hours), depending on the parameters implemented in the uncaging experiments (with longer duration for lower nutrient backgrounds).

### Nutrient fluxes and background

The number of moles of glutamate released in a single pulse is given by *n* = *c*_0_π*r*_P_^2^*h*, where *r*_P_ = 175 μm and *h* = 500 μm are the radius and height of the columnar release, respectively. The subsequent diffusion of this axisymmetric cylindrical pulse (diffusivity *D*_C_ = 608 μm^2^ s^−1^) is well approximated by a point source spreading with a Gaussian profile *c*(*r*,*t*). Given the cylindrical symmetry of the uncaging process, the nutrient mass can be converted into number of molecules per unit distance (in the axial direction), with the number of molecules released during photolysis calculated as M* = *N*_A_*n*/*h* = 2.3 × 10^9^ μm^−1^, where *N*_A_ is Avogadro’s constant. The average flux of nutrient injected into the bacterial arena is calculated as ϕ = M*/ (*N*_A_*A*Δ*t*), where *A* = 1 cm^2^ is the area of the bacterial arena over which the chemical pulses are released. The continuous flow of the artificial seawater solution of 1 mM MNI-caged-l-glutamate in the washing channel above the PCTE membrane replenishes the caged compound solution by molecular diffusion and, at the same time, removes the spent medium from the bacterial arena. This maintains the nutrient background, *c*_B_, in a nonequilibrium steady state. This allows for a prolonged series of nutrient pulse releases, but in a manner that (i) avoids any long-term changes in the background chemical profile (i.e., statistical steady state) and (ii) does not require the use of any fluid flow in the arena, which would influence the chemical pulses and bacterial trajectories. The nutrient background is calculated as *c*_B_ = M*τ/(*N*_A_*A*Δ*t*), where τ is the timescale for washing provided by the nanoporous PCTE membrane. The washing timescale of the bacterial arena was characterized by the following standard method: The bacterial arena was first filled with a solution of 100 μM fluorescein in deionized water. The reduction in fluorescence was quantified over time as the channel was exposed to flow of deionized water, using a syringe pump (Harvard Apparatus PHD) at the constant flow rate *Q* used in the experiments (*Q* = 50 μl min^−1^). Images of the arena were recorded every minute during 60 min with an inverted epifluorescence microscope (Nikon Eclipse Ti-E) equipped with an sCMOS camera (Andor Zyla 5.5) using a 4× objective (NA, 0.13). For each pixel at position (*x*,*y*) in the image, we quantified the fluorescein concentration over time, *c*(*x*,*y*,*t*), where *c* is the measured fluorescence intensity. We then computed the spatial average of the concentration *c*_m_(*t*) = <*c*(*x*,*y*,*t*)>*_A_* over the spatial extent of the bacterial arena *A*. The temporal evolution of the fluorescein concentration field was used to compute the decay constant fitted by an exponential function *c*_m_(*t*) = *c*_m_(0) *e*^−*t*/τ^, which sets the washing timescale of the arena in our experimental setup at that specific flow rate (τ = 19.5 min).

### Nutrient field

The chemoattractant profile, *c*(*x*,*y*,*t*), over the bacterial arena was reconstructed numerically by inserting the known series of point sources {*p*_1_,…,*p_n_*} executed in the experimental routine at locations {(*x*_1_,*y*_1_,*t*_1_),…,(*x_n_*,*y_n_*,*t_n_*)} into the 2D diffusion equation. Leaking boundaries at the edges of the bacterial arena were imposed to simulate an infinite domain—reflecting the fact that in the experiments, the uncaging arena is embedded within a larger chamber—and with the experimentally derived washing timescale τ imposed by the flow of fresh medium in the washing channel as a sink term.

### Chemotaxis behavior

For the chemical concentration of DOM given by the smooth function *c*(*x*,*y*,*t*), a bacterium with velocity **v** and position (*x*,*y*) at time *t* performs a noisy measurement of the concentration change in its reference frame, ∂*c*/∂*t* = *N*(*c*_1_, σ_c_^2^). This stochastic measurement is normally distributed with mean *c*_1_ = **v**·∇*c*, and standard deviation σ_c_ = χ *c*(*x*,*y*,*t*)^1/2^, with the sensing factor χ = Π[3/(π*aDT*^3^)]^1/2^, and therefore directly incorporates the fundamental limit of precision with which a cell can measure the gradient. Here Π > 1 is the precision factor, which quantifies the ratio of detection sensitivity to that of a perfect absorber (the accuracy of which is only limited by molecular encounters). The parameter *a* = 0.66 μm is the cell’s radius, *D*_C_ is the diffusion coefficient of glutamate molecules, and *T* = 0.1 s is the sensory integration timescale, that is, the timescale over which bacteria integrate measurements of encounters with glutamate molecules to estimate the gradient [these parameters have been estimated for the same bacterium in (*26*)]. To map the nutrient regions of the DOM signal where the gradients are detectable by bacteria of *V. anguillarum*, we imposed the condition SNR = *c*_1_/σ_c_ > 1, where we simplify the analysis by taking the maximum of the signal *c*_1_max_ = *v* |∇*c*| that a cell detects by swimming directly up the gradient (see the Supplementary Materials).

## Supplementary Materials

### This PDF file includes:

Supplementary Methods Table S1 Figs. S1 to S9 References
