# Supplementary material for: Microscale patchiness controls resource acquisition in populations of foraging bacteria
Source: Sci Adv. 2026 Nov 13;12(46):eaea5399. doi: 10.1126/sciadv.aea5399 (PMC13763866; doi:10.1126/sciadv.aea5399)
Supplement: Supplementary file 1 [file sciadv.aea5399_sm.pdf]

Science Advances
AAAS

Supplementary Materials for

Microscale patchiness controls resource acquisition in populations of foraging bacteria

Francesco Carrara *et al.*

Corresponding author: Douglas R. Brumley, d.brumley@unimelb.edu.au; Roman Stocker, romanstocker@ethz.ch

Sci. Adv. **12**, eaea5399 (2026)
DOI: 10.1126/sciadv.aea5399

This PDF file includes:

Supplementary Methods
Table S1
Figs. S1 to S9
References

Supplementary Methods

Patch scaling. The diffusion process subsequent to the uncaging event of the axisymmetric cylindrical pulse is well-approximated by a point source spreading with a Gaussian profile. The Green's function of a diffusing chemical pulse in 2D with cylindrical symmetry is given by $G(r,t) = (4\pi D(t+T_0))^{-1}\exp[-r^2/(4D(t+T_0))]$, where D is the glutamate diffusivity ($D = 608$ μm^2 s^{-1}) and $T_0 \sim 1$s is the time delay to approximate the cylindrical pulse released in the experiments with a pure Gaussian function. The approximation of the columnar release with the Green's function provided above was tested in ref. (*26*), for a similar radius r_P of the chemical pulse. To obtain the full dimensional solution of a spreading pulse $c_P(r,t)$ as a function of radial distance from its center, r, and time since activation, t, we multiply the function $G(r,t)$ by the number of molecules M released in a pulse, $c_P(r,t) = M\,G(r,t)$. Given the cylindrical symmetry of the uncaging process, the nutrient mass is converted into number of molecules per unit of length, therefore the number of molecules released during photolysis is calculated as $M^* = N_A c_0 \pi r_P^2 = 2.3 \times 10^9$ μm^{-1}, where N_A is Avogadro constant, $c_0 = 35$ μM is the initial concentration of glutamate molecules within each pulse, and $r_P = 175$ μm is the radius of the columnar release.

Bacteria perform chemotaxis by temporal gradient sensing. The value of the ramp rate c_1, the perceived rate of change in local ligand concentration, measured over a short time interval T for a bacterium swimming with swimming velocity $\boldsymbol{v}$ can be approximated by $c_1 = \partial c/\partial t + \boldsymbol{v}\cdot\nabla c$, provided the chemical concentration of DOM is given by the smooth function $c(x,y,t)$. A cell performs a noisy measurement of the concentration change in its own moving reference frame, with standard deviation $\sigma_c = \Pi[3c(x,y,t)N_A/(\pi a D T^3)]^{1/2}$, where $\chi=\Pi[3N_A/(\pi a D T^3)]^{1/2}$ is the chemotactic sensing factor, $a = 0.66$ μm is the cell's radius, $D = 608$ μm^2 s^{-1} is the diffusion coefficient of glutamate molecules, and $T = 0.1$ s is sensory integration timescale, that is the timescale over which a bacterium measures the gradient (see refs. (*26*, *31*, *33*)). The signal-to-noise ratio (SNR) for a swimming bacterium is calculated as $SNR = c_1/\sigma_c$. To map the nutrient regions of the DOM signal where the gradients are detectable by bacteria of *V. anguillarum*, we impose the condition $SNR > 1$. Note that the ramp rate $c_1 = \partial c/\partial t + \boldsymbol{v}\cdot\nabla c$ involves both the temporal derivative and the spatial derivative of the concentration field $c(r,t)$. The expression for the signal-to-noise ratio SNR associated with a chemical pulse, as a function of radial distance, r, from the center of the pulse and time of activation spreading into a chemical background c_B is then

$$SNR(r,t) = M\,(\partial G/\partial t + \boldsymbol{v}\cdot\nabla G)/(\chi[(M\,G + c_B)]^{1/2}). \qquad \text{Eq. 1}$$

To characterize the spatial and temporal extent of a single pulse, we defined the maximal radius $r^*(SNR = 1)$ and lifetime $t^*(SNR = 1)$ associated with the bacterial chemotaxis response. Beyond these values, $SNR<1$, and so no chemotaxis is possible. For the function $G(r,t)$ describing a spreading pulse, its temporal derivative $\partial G/\partial t = G[r^2/(4Dt^2) - t^{-1}]$ can be neglected because its contribution is much smaller, $\varepsilon = \partial G/\partial t \,/\, \boldsymbol{v}\cdot\nabla G = 2D/(v\,r^*) \sim 10^{-2}$, than the magnitude of the spatial term $\boldsymbol{v}\cdot\nabla G = v\,G\,r/(2Dt)$. In order to check the magnitude of ε, we note that most distributions of swimming speeds for the bacterial strains in the ocean, including *Vibrio anguillarum* 12B09 investigated here, sit in the range 25-100 μm s^{-1} (47) (Fig. S7), and typical maximal radii r^* for patches associated with chemical pulses created through photolysis range from a few hundred micrometers to a few millimeters (Figs. S3,S4), depending on the magnitude of the nutrient mass M (Fig. S4) and chemical background c_B (Fig. S3). At diffusivities associated with small molecules such as amino acids ($D \sim 10^2$–10^3 μm^2 s^{-1}), we obtain $\varepsilon \sim$

$10^3/(10^2 \times 10^3) \sim 10^{-2}$. Therefore, the ramp rate measured by a bacterium swimming far away from the center of the pulse is given by $c_1 \approx Mv\nabla G$, where we further simplified the analysis by dropping the dot product and taking the maximum of the signal $v\nabla G$ that a cell detects by directly swimming up the gradient at speed v. The maximal radius r^* and lifetime t^* of the patch are computed by performing the partial derivatives of the SNR with respect to time and radius respectively and imposing them equal to zero, $(\partial SNR/\partial t, \partial SNR/\partial r) = (0, 0)$. For negligible background $c_B \ll c(r, t)$, $\partial SNR/\partial t = 0$ results in the scaling $r^*_L = (12Dt^*)^{1/2}$. By substituting this scaling relationship into the condition $SNR(r^*, t^*) = 1$, the lifetime of the patch can be obtained

$$t^*_L = (3M/\pi e^3)^{1/2}\, v/(2D\chi). \quad \text{Eq. 2}$$

For background $c_B \gg c(r, t)$ it follows from a similar argument that $r^*_B = (8Dt^*)^{1/2}$, and by substituting this scaling relationship into the condition $SNR(r^*,t^*) = 1$, one obtains $Mv\nabla G(r^*_B,t^*_B)c_B^{-1/2} = \chi$. By solving this equation for the time variable t^*_B, it follows that

$$t^*_B = 1/2[M^2v^2/(c_B D^3\chi^2\pi^2 e^4)]^{1/3}, \quad \text{Eq. 3}$$

which shows the scaling $t^*_B \propto M^{1/3}$ in the case where the signal is dominated by background, after the substitution $c_B = M\tau/(A\Delta t N_A)$. Correspondingly, through the condition $\partial SNR/\partial r = 0$ and imposing $SNR = 1$, we can compute the other extreme point t^{max} that is the point in time where the patch finally dissolves (but does not coincide with the maximal radius r^* of the patch as for the lifetime t^*)

$$t^{max} = (M/\pi e)^{1/2}\, v/(2D\chi). \quad \text{Eq. 4}$$

Note that $t^{max} > t^*$ by definition and that there is a simple proportionality relationship $t^{max}/t^* = 3^{1/2}/e$ between the two quantities. The corresponding radius associated with t^{max} can be computed as for r^*, resulting into the scaling $r^{max} = (4Dt^{max})^{1/2}$.

The consumption term $\exp[-t/\tau]$, which is set by the washing rate in our experimental setup, is neglected in first instance from our theoretical analysis because experimentally we are always in the condition for which $t^* \ll \tau$. To verify this assumption, we compute the complete solution (with the consumption term on) by performing a first order expansion $\exp[-t/\tau] \approx (1-t/\tau)$ for negligible background $c_B \ll c(r, t)$ (the case with background $c_B \gg c(r, t)$ follows a similar argument). By inserting this term into Eq. 1 and imposing the condition $SNR(r, t) = 1$, we find that the rescaled lifetime of the patch, including the consumption term, is given at the first order by

$$t^*_\tau = t^*/(t^*/2\tau + 1), \quad \text{Eq. 5}$$

and for the rescaled maximal radius of the patch

$$r^*_\tau = 12Dt^*_\tau(1 + t^*_\tau/3\tau). \quad \text{Eq. 6}$$

The effect of imposing a higher washing rate (that is, a lower timescale τ) corresponds to a shorter patch lifetime t^*_τ, and at the same time a relatively larger maximal radius r^*_τ. We used the simplified expressions of Eqs. 2, 3 to calculate the sensing probability provided in Eqs. 8, 9 and the transition between the two regimes in Eq. 11 (see below "*Sensing probability*"). However, we consider the complete expression of the $SNR(r, t)$ provided in Eq. 1 to compute the numerical solution of r^* and t^* (Figs. S3, S4) that are used for generating the spatial maps of prominence, sensing probability, and relative uptake as a function of nutrient mass M and background c_B in Fig. 4.

Sensing probability. The bacterial sensing probability is computed as the total fraction f_A of the arena where bacteria can perform chemotaxis towards chemical pulses (*SNR* >1). In order to better highlight the two scaling regimes (the low background and the high background), we approximate in the following the space-time extension of a single patch as a disk using the formula $\pi r^{*2} t^*$ (but again we consider the complete numerical expression of f_A for generating the spatial maps as a function of nutrient mass M and background c_B in Fig. 4). Provided that the pulses injected over the bacterial arena A with a frequency Δt^{-1} are randomly distributed, we can compute the sensing probability as (*48*)

$$f_A = 1 - \exp[-\pi r^{*2} t^*/(\Delta t A)], \qquad \text{Eq. 7}$$

which eventually saturates to one for increasing patch densities, $\rho = t^*/(\Delta t A)$ and radius r^*. The average patch inter-distance can be computed as $r_{min} = (2\pi\rho)^{-1/2}$. If $r^* > r_{min}/2$ patches are spatially dense and overlap in space and time. Conversely, when $r^* < r_{min}/2$ patches are well-separated in space and time (this transition is highlighted by the cyan line in Fig. 2B and Fig. 4B,C). At low densities ($\rho\pi r^{*2} \ll 1$), when the patches are not overlapping in space, the probability f_A can be approximated at first order by the expression $f_A \approx \rho\pi r^{*2} = \pi r^{*2} t^*/(\Delta t A)$.

By inserting the scaling relations for r^* and t^* at low background and high background into the expression for f_A, and recalling that $c_B = M\tau/(A\Delta t N_A)$, we find the linear scaling and the sublinear scaling between sensing probability and background c_B (or nutrient flux $\phi = c_B/\tau$) highlighted in Fig. 3A by the magenta and green lines respectively. For the linear regime, by substituting Eq. 2 and the scaling $r^*_L = (12Dt^*)^{1/2}$ into the approximated expression for $f_A \approx \pi r^{*2} t^*/(\Delta t A)$ we find

$$f_A^L = 9M v^2/(e^3\chi^2 D\Delta t A) = 9c_B v^2/(e^3\chi^2 D\tau), \qquad \text{Eq. 8}$$

and by substituting Eq. 3 and the scaling $r^*_B = (8Dt^*)^{1/2}$ for the sublinear regime into Eq. 7 we find

$$f_A^B = 1 - \exp[-(2c_B^{1/3}M^{1/3}v^{4/3})/(e^{8/3}\pi^{1/3}\chi^{4/3}D\tau)]. \qquad \text{Eq. 9}$$

In the linear regime, the sensing probability f_A^L is constant over simultaneous modulation of nutrient mass M and frequency of injection Δt^{-1} (by keeping the background constant). In other words, $f_A^L = 9\phi v^2/(e^3\chi^2 D)$ only depends on the nutrient flux ϕ and not on how the nutrient is distributed in space, that is M, and time Δt. Conversely, in the background sensing regime, by taking a first order approximation in Eq. 9 at low patch density ($\rho\pi r^{*2} = k$, with $k < 1$), we highlight the 1/3 scaling $f_A^B \propto c_B^{1/3}M^{1/3}$ that generates an inverse proportionality relation between M and c_B (white dotted line with slope –1 in Fig. 4B)

$$c_B = k(D\tau)^3\chi^4 e^8\pi/(Mv^4), \qquad \text{Eq. 10}$$

at fixed values of sensing probability, $f_A^B = k$. For $k = ½$, we note that this isocline coincides with the cyan line delimiting the regime with overlapping patches ($r^* = r_{min}/2$) in Fig. 4B.

We can compute the transition between the two regimes by equating the two expressions of the sensing probability $f_A^L = f_A^B$ in Eqs. 8 and 9 (and applying a first order approximation for the exponential function in Eq. 9 at low patch density). The transition occurs at values of background (white dotted line with slope ½ in Fig. 4B)

$$c_B = (2^{1/2}/3)^3(Me/\pi)^{1/2}\chi/v, \qquad \text{Eq. 11}$$

where we highlight the $c_B \propto M^{1/2}$ scaling. This follows as a consequence of the definition of the signal-to-noise ratio *SNR* (Eq. 1). This transition between linear and background regime corresponds also to the patch prominence, ε_M, defined as the ratio of nutrient remained in a pulse at (r^*,t^*) over background $c_P(r^*,t^*)/c_B$ dropping below one (see white dotted line with slope ½ in Fig. 4A). The transition can be derived by imposing $\varepsilon_M = 1$ for the background sensing case (with an equivalent derivation for the linear sensing provided we substitute the according expressions), with $c_P(r^*,t^*) = MG(r^*_B,t^*_B) = M(4\pi Dt^*_B)^{-1}e^{-2}$, where we used the background scaling $r^*_B = (8Dt)^{1/2}$. By substituting Eq. 3 for t^*_B and solving for c_B we obtain the same proportionality relationship $c_B \propto M^{1/2}\chi/v$ as in Eq. 11.

Nutrient uptake. To quantify the uptake of a chemotactic population foraging in a patchy landscape, we multiply the sensing probability f_A and the benefit B from a patch. The benefit B is computed by performing the time integration

$$B = \int_{T_0}^{T_U} [M\, G(r^*, t) + c_B]\, dt, \qquad \text{Eq. 12}$$

where $G(r^*,t) = (4\pi Dt)^{-1}\exp[-r^{*2}/(4Dt)]$ is the Green's function of a diffusing chemical pulse in 2D constrained on the solution $r^* \propto t^{1/2}$ imposed by the signal-to-noise-ratio scaling and M is the nutrient mass in a single pulse, and $\boldsymbol{T_0}$ = 1 s is the time delay used for the approximation of the cylindrical solution with the Gaussian function (*26*). This expression assumes that on average, a population of bacteria performing chemotaxis towards a nutrient pulse is exposed to a nutrient load that scales according to the signal-to-noise ratio *SNR*, $r^* \propto t^{1/2}$. This assumption is well grounded on our experimental observations, given the proportionality relationship obtained in Fig. 2B between the bacterial clustering length scale, ξ, and the nutrient length scale, r_C, defined as the minimum between the patch maximal radius and the average inter-patch distance, $r_C = \min[r^*, r_{\min}/2]$. To compute the interaction time $T_I = T_U - T_0$, which corresponds to the duration of the interaction of a bacterium with a chemical pulse created at time T_0, we make the following consideration: if $r^* > r_{\min}/2$ on average patches are interconnected in space, therefore the time of interaction with one chemical pulse is given by its lifetime $T_I = t^*$. Conversely, if the patch density is lower at $r^* < r_{\min}/2$, the time interval is computed as the sum of two contributions: a bacterium would interact with a patch for its lifetime t^* extended by the time to find the next patch $T_I = t^* + t_s$, where t_s is the search time to find the next patch. The search time represents the time that a bacterium spends outside of a patch before the exposure to the next chemical pulse, and is calculated according to the following equation, $t_s = \Delta t(r_{\min}^2 - r^{*2})/r_{\min}^2$, where Δt is the interarrival time between two consecutive chemical pulses. By substituting $G(r^*, t) = M(4\pi Dt^*)^{-1}e^{-1}$ and performing the time integration between T_0 and T_U we obtain $B = M/e \ln[T_U/T_0] + c_B T_I$. To obtain the uptake relative to the potential uptake of a non-chemotactic population b_U, we take the ratio $b_U = f_A B/(c_B T_I)$ of the potential uptake by the chemotactic population divided by the potential uptake for a randomly distributed motile population that is exposed to the background c_B for the same interaction time T_I as the chemotactic population. By substituting the expressions for f_A and B, the relative uptake is given by

$$b_U = (1 - \exp[-\pi r^{*2} t^*/(\Delta t A)]) M/(c_B T_I\, e) \ln(T_U/T_0) + 1, \qquad \text{Eq. 13}$$

where $T_I = T_U - T_0$ is the interaction time with a patch computed as above. We used this expression to perform a fit of the relative uptake b_U^E computed experimentally over different nutrient backgrounds in Fig. 3B, that is $b_U^E - 1 = \beta(1 - \exp[-\pi r^{*2} t^*/(\Delta t A)]) M/(c_B T_I\, e) \ln[T_I/T_0]$,

with a fitting parameter $\beta = 1.88$, and then mapped the relative uptake as a function of nutrient mass and background in Fig. 4C. The fitting parameter β represents the compound effect on potential uptake of bacterial chemotaxis and accumulation around chemical pulses in the nutrient landscape (see the intercept of the pair correlation function, $g(r < 100)$ in Fig. S5), whereas for the derivation of the benefit B in the formula provided in Eq. 12, we assumed that the bacterial distribution around a chemical pulse is flat.

Nutrient consumption. According to literature, the maximum uptake of amino acid molecules sits in the range $V_{\max} \in \{1 \times 10^4 - 1 \times 10^5\}$ s^{-1} , and typical affinities for amino acids for copiotrophic strains sit in the range $K_M \in \{0.5 - 5\}$ μM (*49–51*). At values of $V_{\max} = 5 \times 10^4$ s^{-1} and $K_M = 2$ μM – in the middle of these ranges – the local uptake timescale for bacteria with concentration $c_{bact} \sim 1 \times 10^7$ cells mL^{-1} can be estimated as $\tau_U = K_M/(V_{\max}\, c_{bact}) = 2.4 \times 10^3$ s. This value of the uptake timescale is larger than the washing timescale imposed in our chamber ($\tau_U = 1.2 \times 10^3$ s) and therefore is not taken into consideration in our theoretical analysis. For additional discussion regarding the effect of nutrient consumption on bacterial chemotactic performance under similar photolysis setup and nutrient field conditions, we refer the interested reader to ref. (*26*), where the assessments were carried out through numerical simulations.

Energy uptake and consumption for a swimming cell. The energy expended on motility for a swimming cell is $E_i = v_i^2\Omega/\eta$, with v_i the speed of a motile cell i, η the efficiency of the flagellum (with a range of 1–2% (*52*)) and Ω the resistance coefficient of the bacterium including its flagellum ($\Omega = 4.1 \times 10^{-8}$ Ns m^{-1} (*19*)). The energy expenditure for a population of swimming cells is on average $E = \Sigma_i E_i /N = 6 \times 10^4$ ATP/s, assuming a conversion factor of 8×10^{-20} J/ATP (*43*), where N is the number of cells captured in each video and E_i the energy spent per cell as above. This motility energy requirement is higher than the typical maintenance energy per time during starvation, estimated at 1×10^4 ATP/s per cell or smaller (*53*, *54*), so for simplicity we do not consider the contribution to the energy budget of this maintenance. To compute the energy intake provided by the glutamate molecules that a cell uptakes (both for the motile and non-motile), we estimate the uptake according to Michaelis-Menten kinetics $U(c) = cV_{\max}(c + K_M)$, with maximum uptake of amino acids molecules, $V_{\max} = 5 \times 10^4$ s^{-1} and affinity $K_M = 2$ μM. An energy conversion equivalent per glutamate molecule of 15 ATP is assumed according to literature (*55*). As a side note, the uptake timescale derived through transport limited uptake as implemented here is approximately two orders of magnitude larger than the diffusion limited uptake timescale $\tau_U{}^D = (4\pi a D c_{bact})^{-1}$, computed by taking the ratio $\tau_U/\tau_U{}^D = (K_M/V_{\max})\, 4\pi a D = 134$, with the cell's radius $a = 0.66$ μm and a diffusion coefficient $D_C = 608$ μm^2 s^{-1}. The diffusion limited uptake timescale would result in $\tau_U{}^D = 18$ s at the same bacterial concentration used in the center of the patch, $c_{bact} = 1 \times 10^7$ cells mL^{-1}. Thus, background concentrations of amino acids in the range $c_B \in \{0.4 - 8\}$ μM as in our experiments would provide an energy intake of about $U(c_B) = 1 \times 10^5$ ATP s^{-1} to 7.5×10^5 ATP s^{-1} that potentially is enough to fuel motility indefinitely. Results for potential uptake as a function of patch density relative to those of a non-motile population (Fig. S6) discount the motility cost for the motile population only. The relative uptake is calculated as $b_U{}^E = N^{-1}\, \Sigma_i(U_i(c) - E_i)/U(c_B)$, where $U_i(c)$ and E_i are the uptake and energy expenditure for the cells i in a population of N swimming cells, and $U(c_B)$ is the average uptake of a randomly distributed non-motile population of cells.

Variable	Symbol	Definition / Value
Experimental arena size	A	1 cm^2
Nutrient diffusivity	D	608 $\mu m^2\ s^{-1}$
Washing timescale	τ	1150 s^{-1}
Interval between pulses	Δt	5—105 s
Nutrient pulse mass	M^*	$2.3\times10^9\ \mu m^{-1}$
Nutrient pulse initial concentration	c_0	35 μM
Background concentration	c_B	$M^*\tau/(A\Delta t N_A)$, nM
Nutrient flux	ϕ	$M^*/(A\Delta t N_A)$, nM s^{-1}
Pulse concentration	c_P	M^*G, nM
Cell body radius	a	0.66 μM
Sensory integration timescale	T	0.1 s
Mean swimming speed	v	47.5 μm s^{-1}
Chemotactic precision	Π	1—10
Chemotactic sensing factor	χ	$\Pi[3N_A/(\pi a D T^3)]^{1/2}$
Mean bacteria concentration	c_{bact}	1×10^7 cells mL^{-1}
Signal to noise ratio	SNR	$[\partial c/\partial t + \boldsymbol{v}\cdot\nabla c]/(\chi\ c^{1/2})$
Patch maximal radius	r^*	$\partial SNR/\partial t = 0\|_{SNR(r,\ t) = 1}$, μm
Patch persistence time	t^*	$\partial SNR/\partial r = 0\|_{SNR(r,\ t) = 1}$, s
Patch prominence	ε_M	$c_P(r^*, t^*)/c_B$
Patch density	ρ	$t^*/(\Delta t A)$, cm^{-2}
Average patch inter-distance	r_{min}	$(2\pi\rho)^{-1/2}$, μm
Sensing probability	f_A	$1-\exp[-\pi r^{*2} t^*/(\Delta t A)]$
Nutrient length scale	r_C	$\min[r^*, r_{min}/2]$, μm
Bacterial clustering length scale	ξ	$\propto r_C$, μm

Table S1: Parameters used throughout experimental procedures and numerical modelling. $N_A = 6.022\times10^{23}$ is the Avogadro number. For the calculation of the pulse concentration $c_P(r,t)$, G is the Green's function of a diffusing chemical pulse in 2D with cylindrical symmetry, given by $G(r,t) = (4\pi D(t+T_0))^{-1}\exp[-r^2/(4D(t+T_0))]$. Here $T_0 \sim 1$s is the time delay to approximate the cylindrical pulse released in the experiments with a pure Gaussian function. The approximation of the columnar release with the Green's function provided above was tested in ref. (*26*), for a similar radius r_P of the chemical pulse.

Supplementary Figures

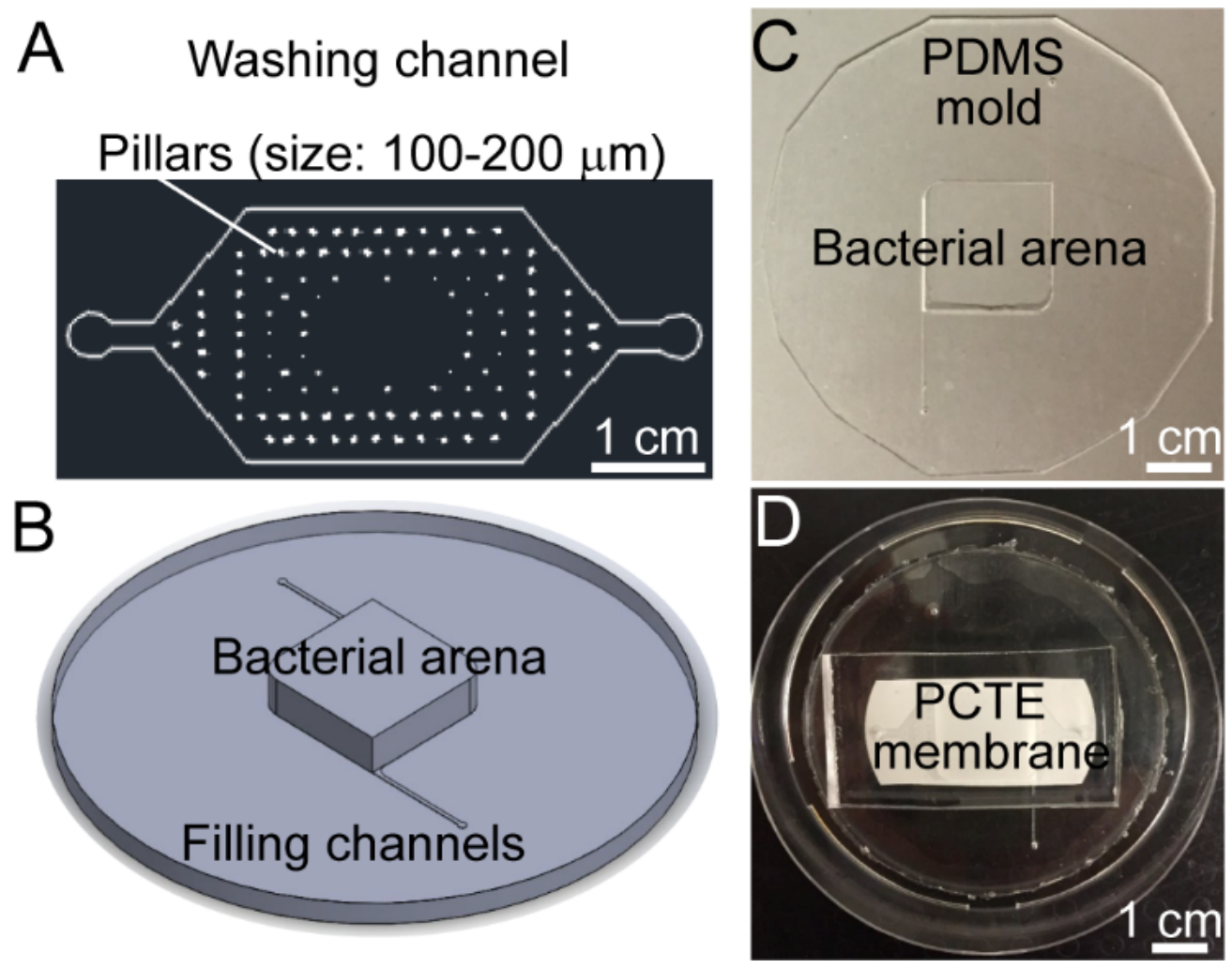

Figure S1. Schematics of the microfluidic device for creating long run dynamic nutrient conditions. (**A**) Mask of the washing channel with embedded microscale pillars (see geometrical pattern with different sizes in the range 100—200 μm, height of the channel 200 μm). (**B**) Design of the 3D printed master used to create the bacterial chamber by casting PDMS. Note the different heights for the bacterial arena (height: 500 μm) in the center and the filling channels (height: 50 μm) to minimize flow in the arena. (**C**) The PDMS layer with the patterning of the bacterial chamber as in panel **B**. Total dimension of the bacterial arena is 2×2 cm^2. (**D**) The complete device (top view), where the polycarbonate PCTE membrane (highlighted in white) is bonded to the top and bottom to the PDMS washing channel and the bacteria arena respectively. The lower surface of the 3D arena is polystyrene, as it is bonded to a Petri dish (60 mm × 25 mm) in order to reduce bacterial attachment compared to, for example, the more commonly used glass slides employed in microfluidics. The flow rate in the washing channel was maintained at 50 μL min^{-1}, which results in a flow speed of 200 μm s^{-1}. However, the presence of the PCTE membrane with 400 nm nanopores prevents any flow in the bacteria arena. Molecular diffusion facilitates the washing (with characteristic time of $\tau = 19.5$ min) of uncaged glutamate and spent metabolic media and at the same time replenishes caged glutamate to achieve a statistical steady state (see Fig. 1B for snapshots of nutrient landscapes and Fig. S2A for integrated nutrient profiles in the bacterial arena). This microfluidic device, in combination with photolysis, allows the rapid and precise releasing of a known mass of chemoattractant in a constant nutrient background to characterize the bacterial response under dynamic and patchy conditions. The chemical pulses occur in a large quasi-2D domain according to a user-defined sequence in space and time that is fully customizable via software (which can be used to impose random sequences or particular patterns). In general, by modulating the focused LED beam in time, amplitude, size, and geometry, a great flexibility can be achieved to generate different types of chemical environments.

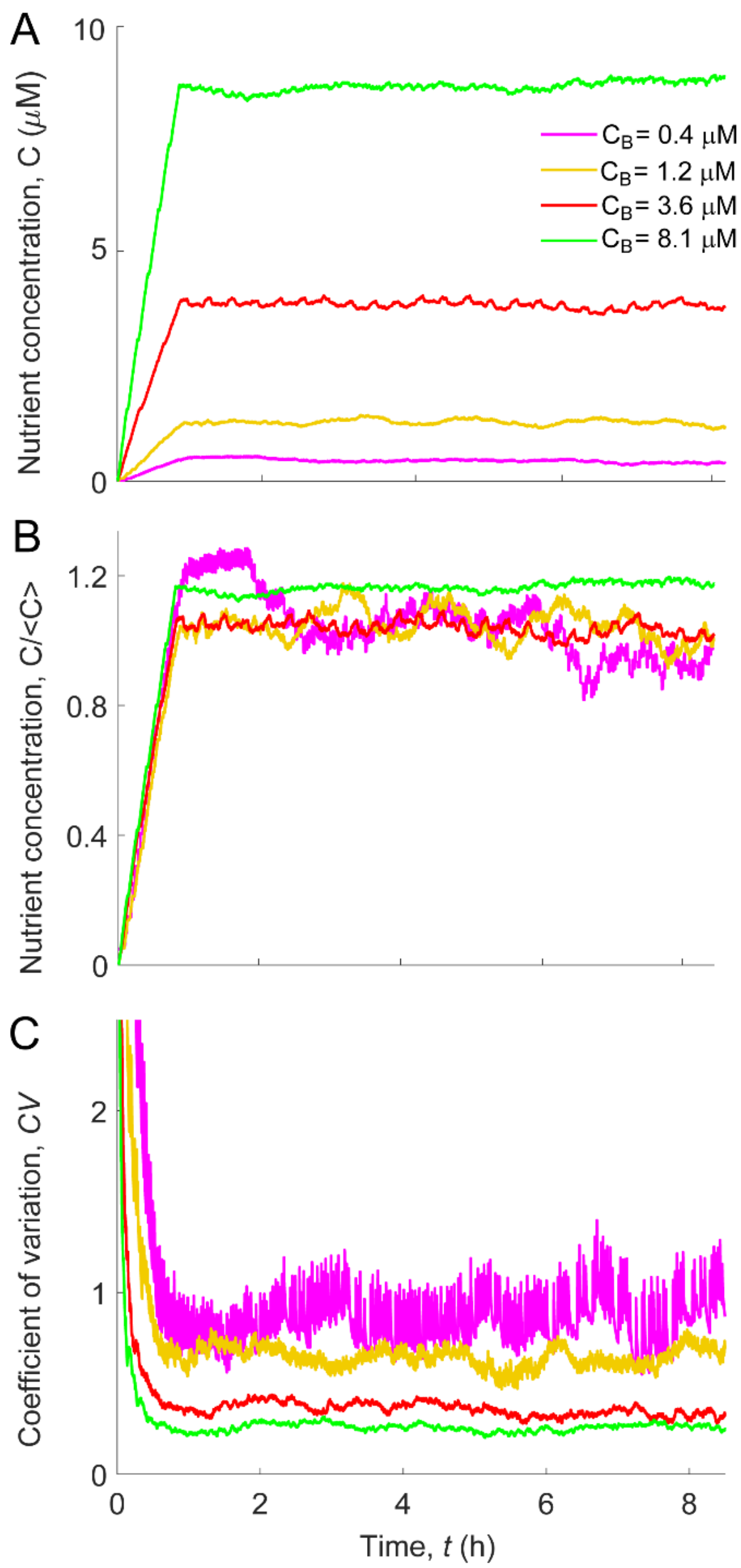

Figure S2. Temporal evolution of the nutrient profiles in the bacterial arena. (**A**) Nutrient concentration at different values of background is computed by integrating the nutrient profile over the whole surface of the bacterial arena (see Fig. 1B for snapshots of the corresponding nutrient landscapes). Temporal fluctuations correspond to the release of chemical pulses by photolysis in the experimental setup and the corresponding dissipation due to the molecular diffusion through the PCTE membrane that sets the washing timescale ($\tau = 19.5$ min). Note the reaching of a statistical steady state (and an associated chemical background c_B) after approximately half an hour of exposure to the experimental treatment. (**B**) Same as in panel **A** but rescaled over the chemical background c_B to highlight the relative magnitude of the fluctuations. (**C**) Temporal evolution of the coefficient of variation (*CV*) calculated as the ratio of the spatial variance (resolution 20×20 μm^2), over the chemical background, c_B, at different levels of chemical background c_B.

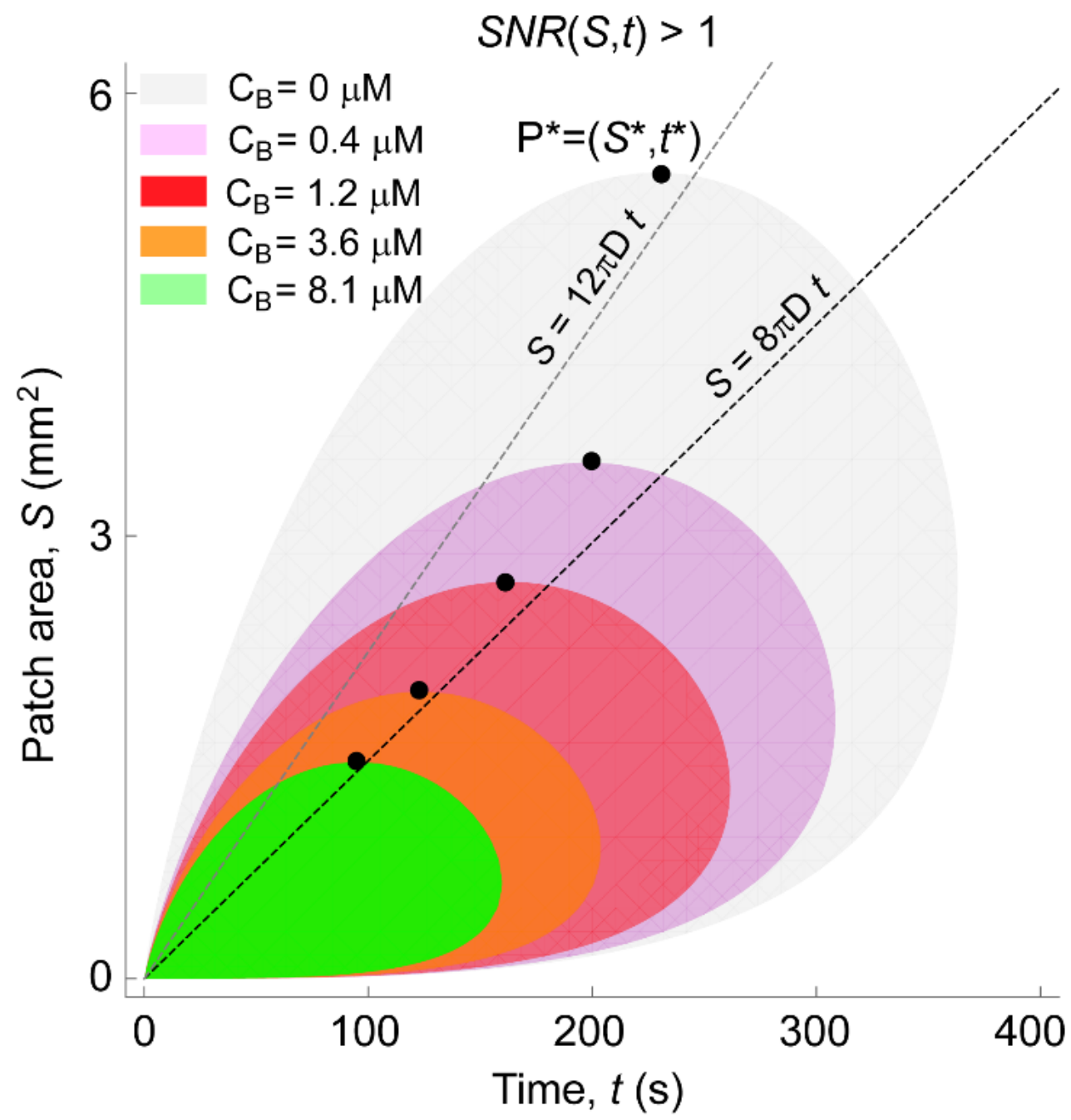

Figure S3. Spatiotemporal scaling of the signal to noise ratio (*SNR*) for a chemical pulse depends on the nutrient background. The patch area associated with the release of the chemical pulse is computed as the region with $SNR(S, t) > 1$, with $S = \pi r^2$ and r is the patch radius. The chemical pulse is modeled as an axisymmetric gaussian (with cylindrical symmetry) with an initial uncaged glutamate mass of $M^*=$ 2.3×10^9 μm^{-1} and diffusion coefficient 608 μm^2 s^{-1}. This mass M^* corresponds to the number of molecules per unit of length released during the uncaging event thorough photolysis (initial glutamate concentration C_0 = 35 μM, radius r_P = 175 μm). The patch expands until a maximal area S^* at time t^* (P*, black dots for different background, c_B) before contracting and eventually disappearing because its *SNR* drops below the value of 1. At this time, the bacterial chemotaxis ceases to be possible according to the sensory noise theory. The maximal patch area S^* grows following two scaling regimes (see black dotted lines) depending on the chemical background against which the chemical pulse is released: at extremely low chemical background (gray color, c_B = 0 μM) the theoretical maximal patch area is calculated as $S^* = 12\pi Dt$, whereas at high chemical backgrounds (green color, c_B = 8.1 μM) $S^* = 8\pi Dt$ (see SI Appendix Supplementary Text). Note that the point P* at zero background calculated numerically has a ratio $S^*/\pi Dt$ slightly larger than 12 because of the effect of the nutrient consumption rate τ imposed by the washing in the channel (see Fig. S4 and Supplementary Methods section at Eqs. 5, 6 for the patch scaling that includes consumption).

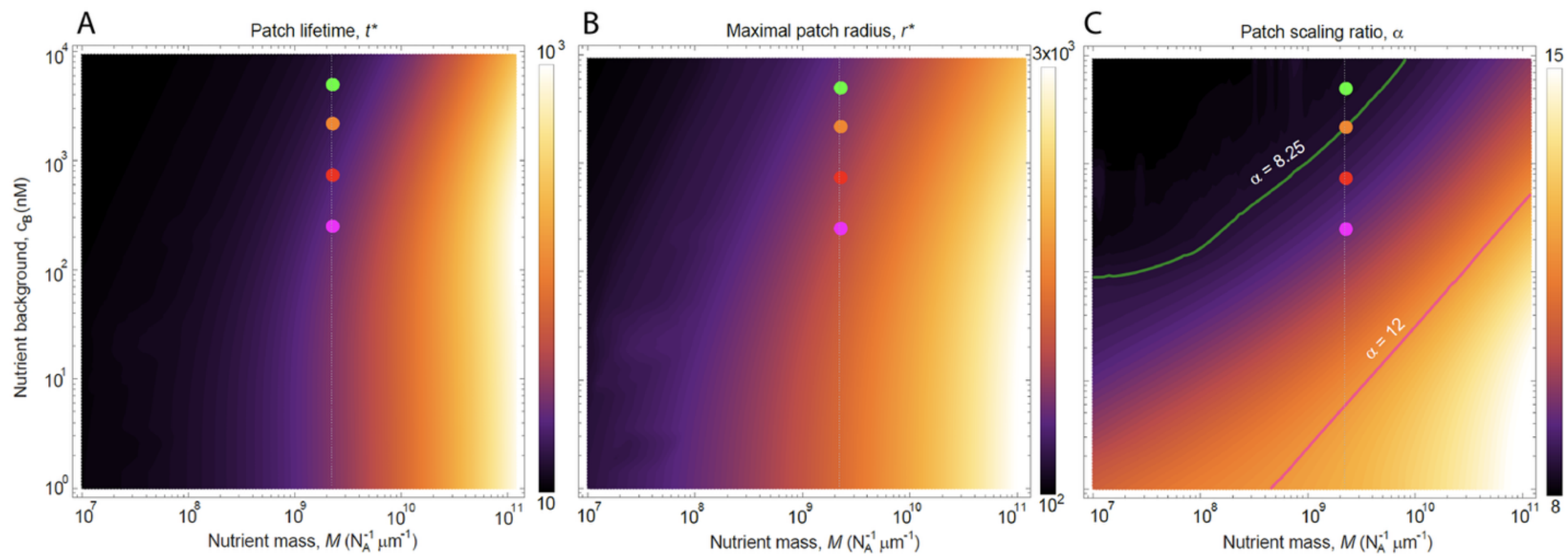

Figure S4. Spatial and temporal scaling of nutrient patches as a function of nutrient mass and nutrient background. The patch lifetime t^* (**A**) and the maximal radius r^* (**B**) associated with a chemical pulse of mass M and nutrient background c_B is computed numerically by extracting the maximum radius and the associated time on the contour line $SNR(r,t) = 1$ associated with the spreading pulse. (**C**) The patch scaling ratio, $\alpha = r^{*2}/(Dt)$, is calculated by dividing the corresponding maximal radius and lifetime calculated in panels (**A**) and (**B**). The maximal patch radius r^* grows following two scaling regimes depending on the chemical background against which the chemical pulse is released: at extremely low chemical background ($c_B < 100$ nM) or for high nutrient mass ($M > 10^9\ \mu m^{-1}$) the theoretical maximal radius is calculated as $r^* = (12Dt^*)^{1/2}$, whereas at high chemical backgrounds ($c_B > 1\ \mu M$) or low nutrient mass ($M < 10^9\ \mu m^{-1}$) the radius scales as $r^* = (8Dt^*)^{1/2}$ (see Supplementary Methods). The green and magenta lines show the isoclines for ratios α corresponding to the background ($\alpha = 8.25$) and the zero background ($\alpha = 12$) regimes respectively. The theoretical values predicted in the absence of consumption would give ratios α of 8 and 12 for these two regimes, respectively. Note that the scaling at zero background calculated numerically has ratios α slightly larger than 12 because of the effect on the scaling of the nutrient consumption timescale τ imposed by the washing in the channel (see Supplementary Methods for the patch scaling that includes consumption at Eqs. 5, 6). Patches with a mass $M \sim 10^7\ \mu m^{-1}$ would correspond approximately to the quantity of nutrient released in the burst of a cyanobacterium with radius $a_C = 1\ \mu m$, whereas $M \sim 10^{11}\ \mu m^{-1}$ to the nutrient released from a large diatom with radius $a_D = 100\ \mu m$. The chemical pulse is modeled as an axisymmetric gaussian (with cylindrical symmetry) with an initial uncaged glutamate molecules mass M and diffusion coefficient 608 $\mu m^2\ s^{-1}$. The mass $M^* = 2.3\times10^9\ \mu m^{-1}$ highlighted by the white dotted line corresponds to the number of molecules per unit of length released during the uncaging event thorough photolysis in the multipatch experiments (initial glutamate concentration $c_0 = 35\ \mu M$, radius $r_P = 175\ \mu m$). Note that these maps show the spatiotemporal scaling of $r^*$and t^* generated from the condition $SNR(r,t) = 1$ of a single patch, whereas panels Fig. 4B,C show the sensing probability for a nutrient landscape with multiple patches deployed with interarrival time Δt over the area A, where $c_B = M\tau/(\Delta t A)$.

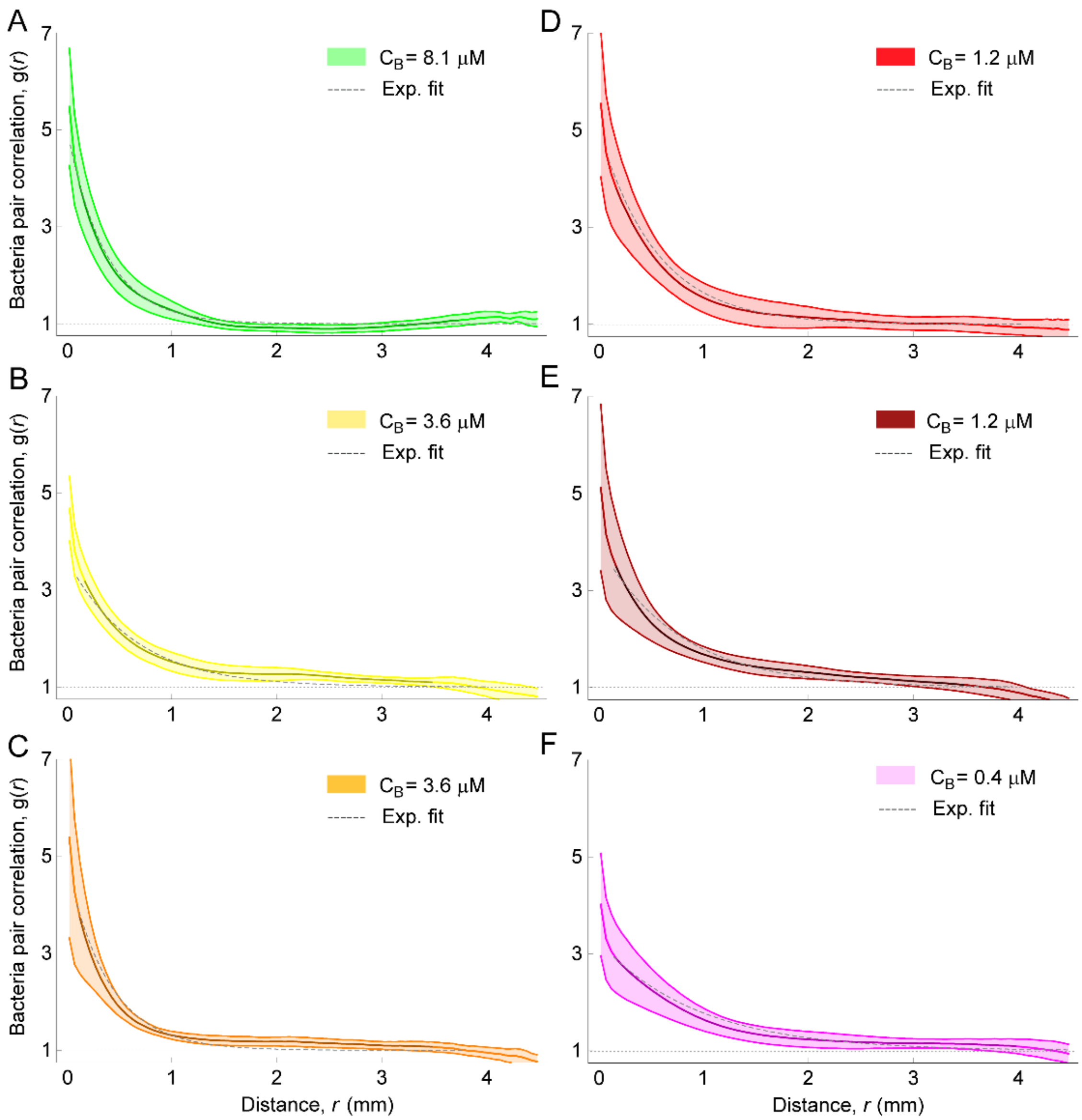

Figure S5. Bacterial accumulation clusters as a function of the chemical background. (**A-F**) Bacterial pair-correlation function $g(r)$ in linear scale (compare to semi log-scale in Fig. 2A) as a function of nutrient background c_B (color coded), where nutrient patchiness was introduced by the release of chemical pulses at the microscale. Dotted lines are exponential fits according to the formula $g(r) \propto e^{-r/\xi}$, with ξ representing the characteristic size of the bacterial clusters (see Fig. 2A) and r is the distance between two bacterial cells. Panels **B**, **C** and **D**, **E** show the pair-correlation function $g(r)$ for the two replicates at c_B = 3.6 μM and c_B = 1.2 μM respectively, whereas panels **A** and **F** show $g(r)$ for the one experiment performed at c_B = 8.1 μM and c_B = 0.4 μM respectively. For each replicate, the confidence interval was obtained through temporal averaging (mean +/- 1 s.d.), with time delay between successive images of the bacterial position in the range 15—30 min depending on the treatment. All plots show an exponential decay for $g(r)$ to the value of one at larger distances, which corresponds to a random distribution of cells as expected by theory.

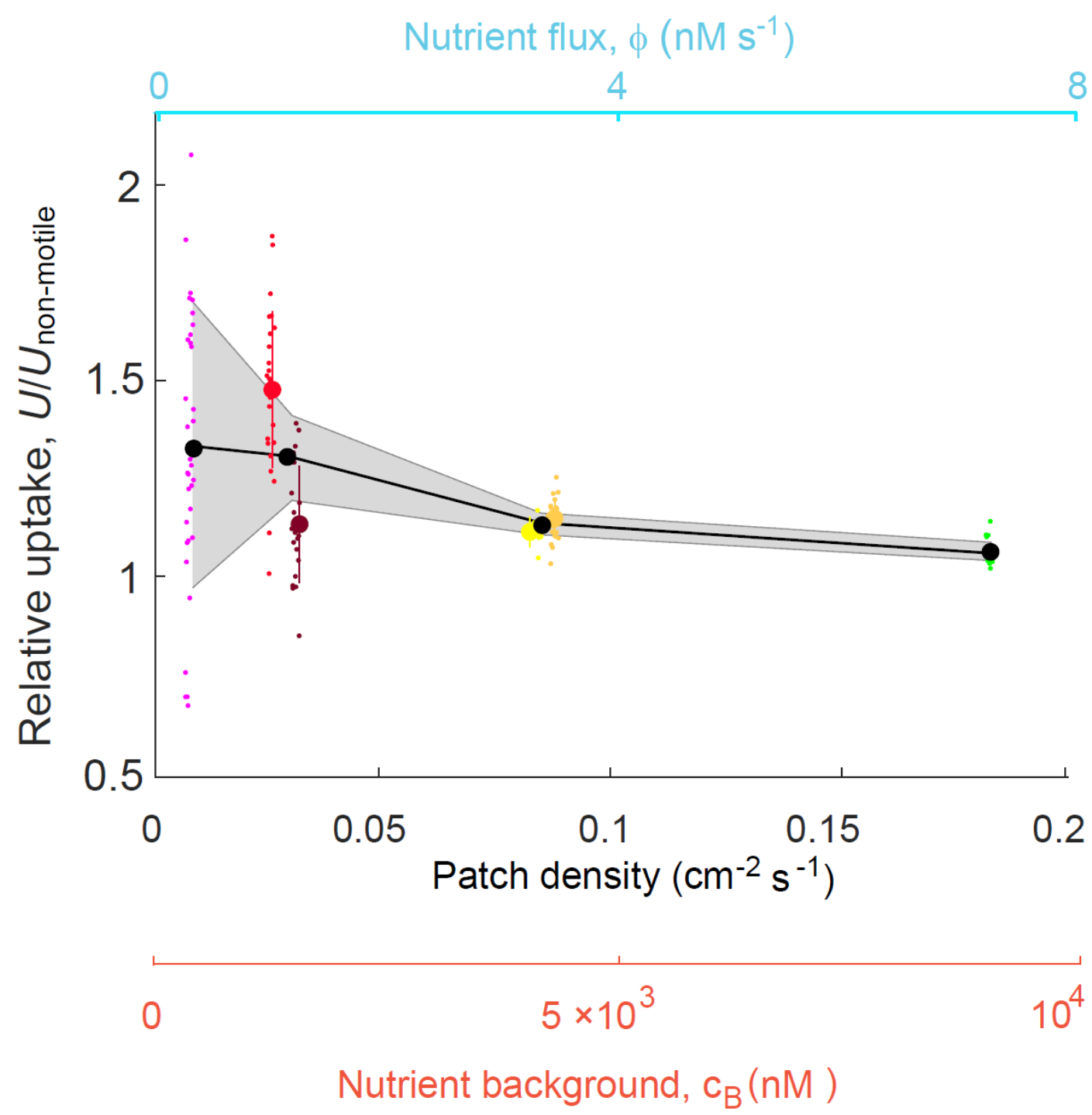

Figure S6. Population nutrient uptake as a function of patchiness relative to a non-motile population. Relative uptake b_U at the population level as a function of patch density is computed by rescaling the potential nutrient uptake at the population level *u* with that of a non-motile population with randomly distributed motile cells (see Supplementary Text). As the patch interarrival time increases (density per unit of area decreases), note the corresponding increase in the relative uptake up to a maximum that occurs between the red and magenta experimental datasets. Compared to the maximum location for the non-chemotactic population presented in Fig. 3B, note the shift towards higher values of patch density, due to the bacterial energy expenditure for the contribution to swimming (see Supplementary Text).

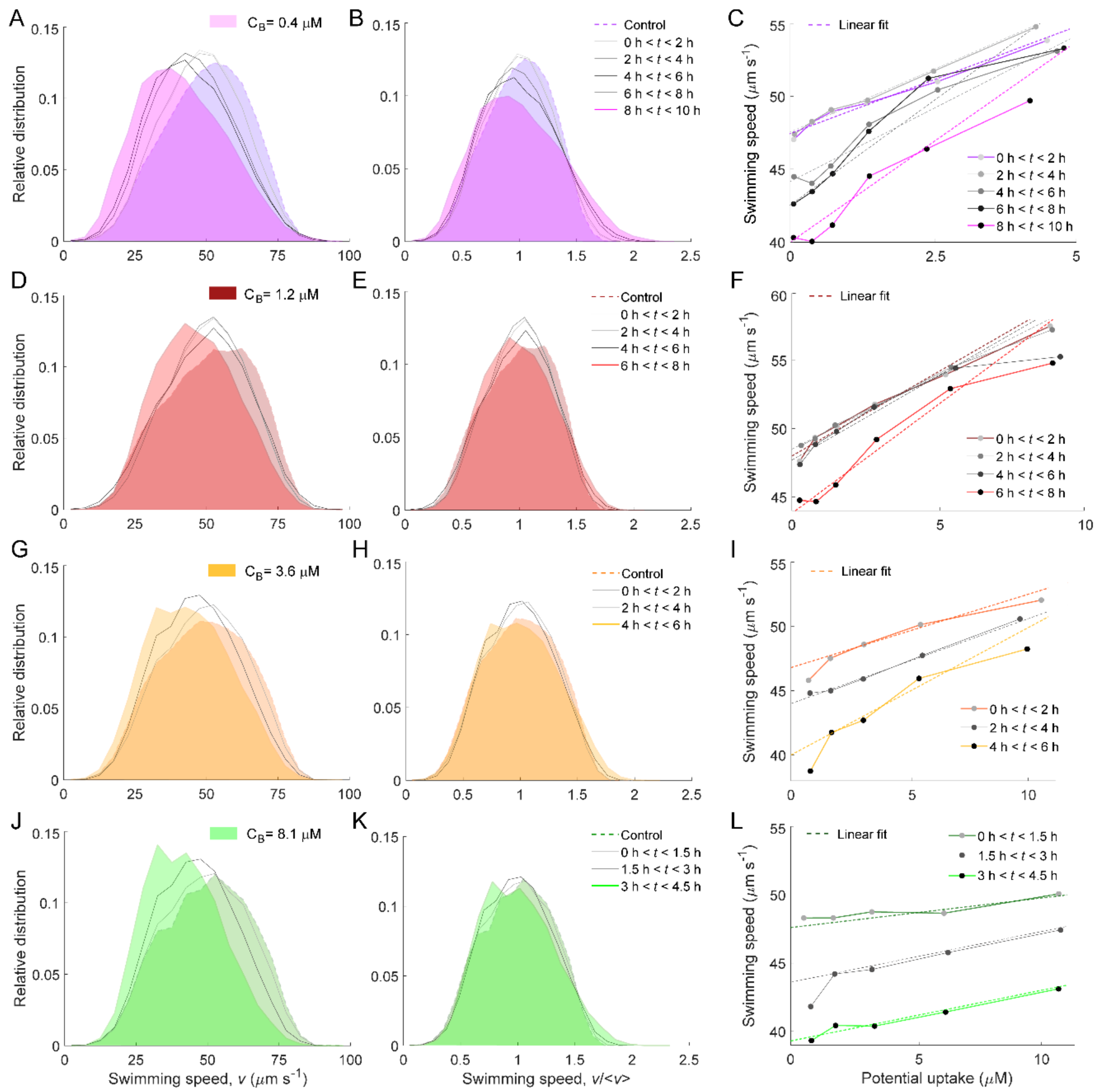

Figure S7. Temporal evolution of swimming speed and potential nutrient uptake as a function of nutrient background in a population of *V. anguillarum*. Relative distribution of swimming speed *v* across a population exposed to pulses at nutrient background values (**A**) c_B = 0.4 μM, (**D**) c_B = 1.2 μM, (**G**) c_B = 3.6 μM, and (**J**) c_B = 8.1 μM. Results as a function of time show a decrease in the mean speed (see color coded lines for control and different time points). Each time bin sums over all trajectories collected over the time points within each replicate. Panels D and G show the average of two replicates whereas A and J show the one replicate at that nutrient background. The mean swimming speed decreases by about 20% over the course of experimental treatments. (**B, E, H, K**) Same as in left column but rescaled over the mean speed <*v*> to highlight the higher moments of the swimming speed distribution. The coefficient of variation $CV_v = \sigma_v/\langle v \rangle$, with σ_v the standard deviation of the swimming speed distribution, increased over time at all backgrounds from about 0.28—0.29 at the beginning of the experiments to 0.31—0.37 at the end. (**C**, **F**, **I**, **L**) Swimming speed as a function of potential uptake shows a positive slope in all the different nutrient backgrounds with a progressive decrease of the magnitude of the slope at increasing background concentrations (c_B = 0.4 μM: 1.38 ± 0.02; c_B = 1.2 μM: 0.86 ± 0.01; c_B = 3.6 μM: 0.42 ± 0.02; c_B = 8.1 μM: 0.21 ± 0.01). Results are given in units of μm s^{-1} μM^{-1} and aggregated over time (dotted lines are the linear fit, dots are experimental data). We also detected a progressive increase of the slope over time (c_B = 0.4 μM: 1.0 ± 0.03 at 0 < *t* < 2 h, 1.72 ± 0.05 at 8 < *t* < 10 h; c_B = 1.2 μM: 0.83 ± 0.02 at 0 < *t* < 2 h, 1.05 ± 0.03 at 6 < *t* < 8 h; c_B = 3.6 μM: 0.36 ± 0.02 at 0 < *t* < 2 h, 0.45 ± 0.02 at 4 < *t* < 6 h; c_B = 8.1 μM: 0.14 ± 0.01 at 0 < *t* < 1.5 h, 0.24 ± 0.01 at 3 < *t* < 4.5 h) upon exposure to nutrient pulsing (see color-coded lines at different time points).

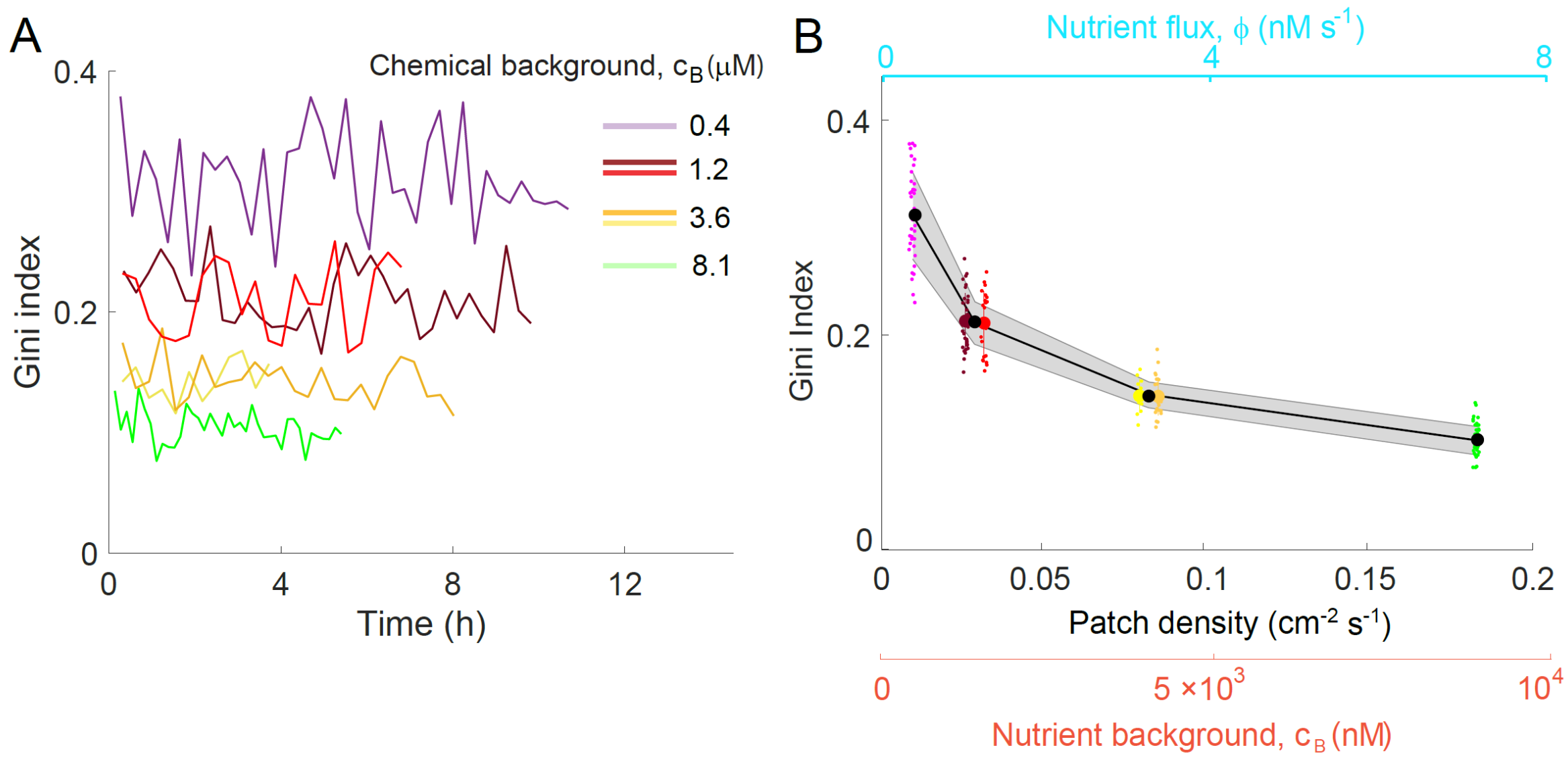

Figure S8. Potential nutrient uptake distribution shows uptake inequality at the population level as a function of patchiness. (**A**) Gini Index as a function of time and (**B**) patch density. The Gini Index increases by increasing the time between the release of two consecutive patches (lowering the patch density and therefore the nutrient flux, see cyan x-axis on top) in a population of *V. anguillarum* foraging in dynamic and heterogeneous landscapes. A higher value of Gini Index, which is bounded between 0 and 1, corresponds to a higher inequality of uptake in the bacterial population. For the calculation of the Gini Index, we adopted the standard formula $G_I = \sum_{i=1}^{N}\sum_{j=1}^{N}\left|u_i - u_j\right| / (\sum_{i=1}^{N} u_i)$, where N is the number of cells in the population, and u_i and u_j are the potential uptake for the cells i and j respectively.

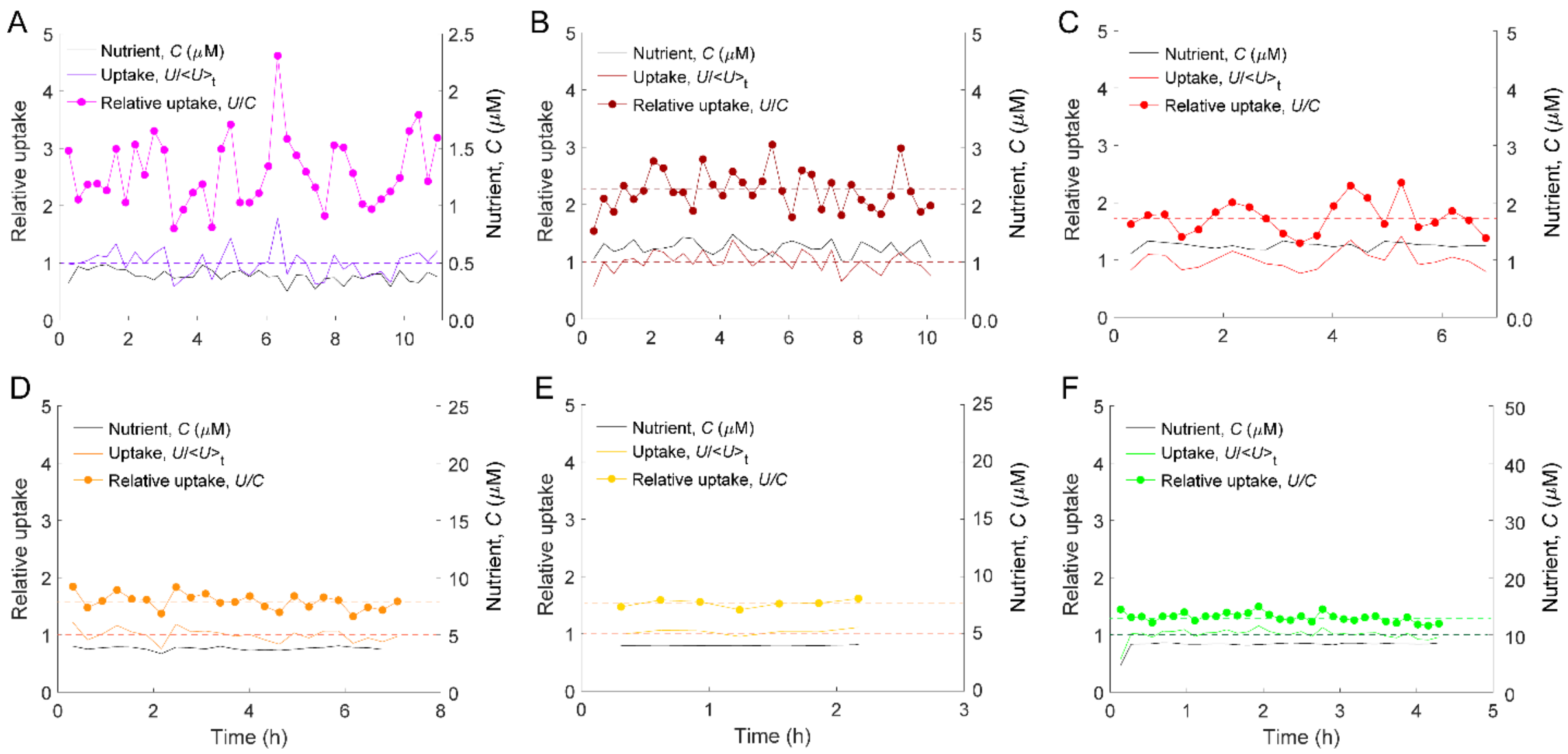

Figure S9. Population uptake remains constant over time upon exposure to microscale patchiness across different nutrient backgrounds. Relative uptake for all the different nutrient backgrounds (dotted lines with experimental points) is computed as the ratio between the observed population uptake divided by the uptake of a randomly distributed population of swimming cells (see also Fig. 3B and Fig. 5C). Fluctuations in nutrient uptake emerge because of the stochastic nature of the foraging process under microscale patchiness. However, the population uptake U divided by the mean uptake $\langle U\rangle_t$ over the entire duration of the experiment remains constant as a function of time (dotted lines fluctuate around $U/\langle U\rangle_t = 1$, color-coded for different nutrient background c_B). Panels **B**, **C** and **D**, **E** show the population uptake for the two replicates at c_B = 1.2 μM and c_B = 3.6 μM respectively, whereas panels **A** and **F** show uptake for the one experiment performed at c_B = 0.4 μM and c_B = 8.1 μM respectively. In all panels, black lines show the average nutrient level C in the bacterial arena over time.

REFERENCES

1. P. Chesson, General theory of competitive coexistence in spatially-varying environments. *Theor. Popul. Biol.* **58**, 211–237 (2000).

2. D. Grünbaum, The logic of ecological patchiness. *Interface Focus* **2**, 150–155 (2012).

3. A. Celani, M. Vergassola, Bacterial strategies for chemotaxis response. *Proc. Natl. Acad. Sci. U.S.A.* **107**, 1391–1396 (2010).

4. E. Kussell, S. Leibler, Phenotypic diversity, population growth, and information in fluctuating environments. *Science* **309**, 2075–2078 (2005).

5. I. Hanski, O. Ovaskainen, The metapopulation capacity of a fragmented landscape. *Nature* **404**, 755–758 (2000).

6. M. E. Ritchie, *Scale, Heterogeneity, and the Structure and Diversity of Ecological Communities* (Princeton Univ. Press, 2009).

7. N. Blackburn, T. Fenchel, J. Mitchell, Microscale nutrient patches in planktonic habitats shown by chemotactic bacteria. *Science* **282**, 2254–2256 (1998).

8. R. Stocker, Marine microbes see a sea of gradients. *Science* **338**, 628–633 (2012).

9. J. P. Armitage, J. M. Lackie, *Biology of the Chemotactic Response* (Cambridge Univ. Press, 1990) vol. **46**.

10. F. Azam, F. Malfatti, Microbial structuring of marine ecosystems. *Nat. Rev. Microbiol.* **5**, 782–791 (2007).

11. A. Buchan, G. R. LeCleir, C. A. Gulvik, J. M. Gonzalez, Master recyclers: Features and functions of bacteria associated with phytoplankton blooms. *Nat. Rev. Microbiol.* **12**, 686–698 (2014).

12. Y. Yawata, F. Carrara, F. Menolascina, R. Stocker, Constrained optimal foraging by marine bacterioplankton on particulate organic matter. *Proc. Natl. Acad. Sci. U.S.A.* **117**, 25571–25579 (2020).

13. P. W. Boyd, H. Claustre, M. Levy, D. A. Siegel, T. Weber, Multi-faceted particle pumps drive carbon sequestration in the ocean. *Nature* **568**, 327–335 (2019).

14. F. Azam, Microbial control of oceanic carbon flux: The plot thickens. *Science* **280**, 694–696 (1998).

15. J. E. Segall, S. M. Block, H. C. Berg, Temporal comparisons in bacterial chemotaxis. *Proc. Natl. Acad. Sci. U.S.A.* **83**, 8987–8991 (1986).

16. A. J. Waite, N. W. Frankel, Y. S. Dufour, J. F. Johnston, J. Long, T. Emonet, Non-genetic diversity modulates population performance. *Mol. Syst. Biol.* **12**, 895 (2016).

17. H. H. Mattingly, K. Kamino, B. B. Machta, T. Emonet, *Escherichia coli* chemotaxis is information limited. *Nat. Phys.* **17**, 1426–1431 (2021).

18. X. Zhang, G. Si, Y. Dong, K. Chen, Q. Ouyang, C. Luo, Y. Tu, Escape band in *Escherichia coli* chemotaxis in opposing attractant and nutrient gradients. *Proc. Natl. Acad. Sci. U.S.A.* **116**, 2253–2258 (2019).

19. J. R. Taylor, R. Stocker, Trade-offs of chemotactic foraging in turbulent water. *Science* **338**, 675–679 (2012).

20. G. M. Whitesides, E. Ostuni, S. Takayama, X. Jiang, D. E. Ingber, Soft lithography in biology and biochemistry. *Annu. Rev. Biomed. Eng.* **3**, 335–373 (2001).

21. K. Son, D. R. Brumley, R. Stocker, Live from under the lens: Exploring microbial motility with dynamic imaging and microfluidics. *Nat. Rev. Microbiol.* **13**, 761–775 (2015).

22. M. M. Salek, F. Carrara, J. Zhou, R. Stocker, J. Jimenez-Martinez, Multiscale porosity microfluidics to study bacterial transport in heterogeneous chemical landscapes. *Adv. Sci.* **11**, e2310121 (2024).

23. F. Carrara, D. R. Brumley, A. M. Hein, Y. Yawata, M. M. Salek, K. S. Lee, E. Sliwerska, S. A. Levin, R. Stocker, Generating controlled, dynamic chemical landscapes to study microbial behavior. *J. Vis. Exp.*, e60589 (2020).

24. L. Xie, C. Lu, X.-L. Wu, Marine bacterial chemoresponse to a stepwise chemoattractant stimulus. *Biophys. J.* **108**, 766–774 (2015).

25. S. Khan, J. L. Spudich, J. A. McCray, D. R. Trentham, Chemotactic signal integration in bacteria. *Proc. Natl. Acad. Sci. U.S.A.* **92**, 9757–9761 (1995).

26. D. R. Brumley, F. Carrara, A. M. Hein, Y. Yawata, S. A. Levin, R. Stocker, Bacteria push the limits of chemotactic precision to navigate dynamic chemical gradients. *Proc. Natl. Acad. Sci. U.S.A.* **116**, 10792–10797 (2019).

27. A. M. Hein, B. T. Martin, Information limitation and the dynamics of coupled ecological systems. *Nat. Ecol. Evol.* **4**, 82–90 (2020).

28. O. X. Cordero, H. Wildschutte, B. Kirkup, S. Proehl, L. Ngo, F. Hussain, F. Le Roux, T. Mincer, M. F. Polz, Ecological populations of bacteria act as socially cohesive units of antibiotic production and resistance. *Science* **337**, 1228–1231 (2012).

29. J. Davis, R. Benner, Quantitative estimates of labile and semi-labile dissolved organic carbon in the western Arctic Ocean: A molecular approach. *Limnol. Oceanogr.* **52**, 2434–2444 (2007).

30. J.-B. Raina, M. Giardina, D. R. Brumley, P. L. Clode, M. Pernice, P. Guagliardo, J. Bougoure, H. Mendis, S. Smriga, E. C. Sonnenschein, M. S. Ullrich, R. Stocker, J. R. Seymour, Chemotaxis increases metabolic exchanges between marine picophytoplankton and heterotrophic bacteria. *Nat. Microbiol.* **8**, 510–521 (2023).

31. A. M. Hein, D. R. Brumley, F. Carrara, R. Stocker, S. A. Levin, Physical limits on bacterial navigation in dynamic environments. *J. R. Soc. Interface* **13**, 20150844 (2016).

32. H. C. Berg, E. M. Purcell, Physics of chemoreception. *Biophys. J.* **20**, 193–219 (1977).

33. T. Mora, N. S. Wingreen, Limits of sensing temporal concentration changes by single cells. *Phys. Rev. Lett.* **104**, 248101 (2010).

34. S. Smriga, V. I. Fernandez, J. G. Mitchell, R. Stocker, Chemotaxis toward phytoplankton drives organic matter partitioning among marine bacteria. *Proc. Natl. Acad. Sci.* **113**, 1576–1581 (2016).

35. F. J. Peaudecerf, R. E. Goldstein, Feeding ducks, bacterial chemotaxis, and the Gini index. *Phys. Rev. E* **92**, 22701 (2015).

36. K. Son, F. Menolascina, R. Stocker, Speed-dependent chemotactic precision in marine bacteria. *Proc. Natl. Acad. Sci. U.S.A.* **113**, 8624–8629 (2016).

37. R. Foffi, D. R. Brumley, F. J. Peaudecerf, R. Stocker, J. Słomka, Slower swimming promotes chemotactic encounters between bacteria and small phytoplankton. *Proc. Natl. Acad. Sci. U.S.A.* **122**, e2411074122 (2025).

38. J. R. Seymour, D. R. Brumley, R. Stocker, J.-B. Raina, Swimming towards each other: The role of chemotaxis in bacterial interactions. *Trends Microbiol.* **32**, 640–649 (2024).

39. T. Kiørboe, U. H. Thygesen, Fluid motion and solute distribution around sinking aggregates. II. Implications for remote detection by colonizing zooplankters. *Mar. Ecol. Prog. Ser.* **211**, 15–25 (2001).

40. S. Gude, E. Pinçe, K. M. Taute, A.-B. Seinen, T. S. Shimizu, S. J. Tans, Bacterial coexistence driven by motility and spatial competition. *Nature* **578**, 588–592 (2020).

41. C. C. Govern, P. R. ten Wolde, Optimal resource allocation in cellular sensing systems. *Proc. Natl. Acad. Sci. U.S.A.* **111**, 17486–17491 (2014).

42. B. Ni, R. Colin, H. Link, R. G. Endres, V. Sourjik, Growth-rate dependent resource investment in bacterial motile behavior quantitatively follows potential benefit of chemotaxis. *Proc. Natl. Acad. Sci. U.S.A.* **117**, 595–601 (2020).

43. J. M. Keegstra, F. Carrara, R. Stocker, The ecological roles of bacterial chemotaxis. *Nat. Rev. Microbiol.* **20**, 491–504 (2022).

44. D. R. Brumley, F. Carrara, A. M. Hein, G. I. Hagstrom, S. A. Levin, R. Stocker, Cutting through the noise: Bacterial chemotaxis in marine microenvironments. *Front. Mar. Sci.* **7**, 527 (2020).

45. N. W. Frankel, W. Pontius, Y. S. Dufour, J. Long, L. Hernandez-Nunez, T. Emonet, Adaptability of non-genetic diversity in bacterial chemotaxis. *eLife* **3**, e03526 (2014).

46. H. H. Mattingly, K. Kamino, J. Ong, R. Kottou, T. Emonet, B. B. Machta, *E. coli* chemosensing accuracy is not limited by stochastic molecule arrivals. *Nat. Phys.* **22**, 123–130 (2026).

47. J. G. Mitchell, L. Pearson, S. Dillon, K. Kantalis, Natural assemblages of marine bacteria exhibiting high-speed motility and large accelerations. *Appl. Environ. Microbiol.* **61**, 4436–4440 (1995).

48. D. R. Cox, V. Isham, *Point Processes* (CRC Press, 1980), vol. **12**.

49. J. Piperno, D. Oxender, Amino acid transport systems in *Escherichia coli* K12. *J. Biol. Chem.* **243**, 5914–5920 (1968).

50. M. Zampieri, M. Hörl, F. Hotz, N. F. Müller, U. Sauer, Regulatory mechanisms underlying coordination of amino acid and glucose catabolism in *Escherichia coli*. *Nat. Commun.* **10**, 3354 (2019).

51. B. Ayo, M. Unanue, I. Azúa, G. Gorsky, C. Turley, J. Iriberri, Kinetics of glucose and amino acid uptake by attached and free-living marine bacteria in oligotrophic waters. *Mar. Biol.* **138**, 1071–1076 (2001).

52. S. Chattopadhyay, R. Moldovan, C. Yeung, X. L. Wu, Swimming efficiency of bacterium *Escherichia coli*. *Proc. Natl. Acad. Sci. U.S.A.* **103**, 13712–13717 (2006).

53. E. Biselli, S. J. Schink, U. Gerland, Slower growth of *Escherichia coli* leads to longer survival in carbon starvation due to a decrease in the maintenance rate. *Mol. Syst. Biol.* **16**, e9478 (2020).

54. C. P. Kempes, P. M. van Bodegom, D. Wolpert, E. Libby, J. Amend, T. Hoehler, Drivers of bacterial maintenance and minimal energy requirements. *Front. Microbiol.* **8**, 31 (2017).

55. C. Kaleta, S. Schäuble, U. Rinas, S. Schuster, Metabolic costs of amino acid and protein production in *Escherichia coli*. *Biotechnol. J.* **8**, 1105–1114 (2013).
